# Experiences and Parents' Perceptions Regarding Dental Interventions Performed on Their Children: A Qualitative Systematic Review

**DOI:** 10.1111/ipd.13318

**Published:** 2025-05-07

**Authors:** Luciana Dalsochio, Anelise Fernandes Montagner, Tamara Kerber Tedesco, Tamires Timm Maske, Françoise Hélène van de Sande

**Affiliations:** ^1^ Graduate Program in Dentistry Universidade Federal de Pelotas – UFPEL Pelotas RS Brazil; ^2^ Graduate Program in Dental Science Universidade de São Paulo ‐ USP São Paulo SP Brazil; ^3^ Graduate Program in Dentistry Universidade Federal do Rio Grande do Sul – UFRGS Porto Alegre RS Brazil

**Keywords:** acceptability, dental care, oral health, paediatric dentistry, perspectives

## Abstract

**Background:**

Understanding the viewpoints of parents of children regarding dental interventions can enhance clinical practices and play a pivotal role in evaluating the acceptability of treatment recommendations.

**Aim:**

To explore the experiences and perceptions of parents regarding minimally invasive dentistry and invasive dental interventions for caries prevention and management in their children.

**Design:**

A systematic search strategy was performed to identify qualitative studies that assessed the phenomenon of interest in October 2022, updated in April 2024. The studies' methodological quality was evaluated, and the final synthesised findings were graded (ConQual). Data were analysed using a meta‐aggregative approach.

**Results:**

Sixteen studies were included. There were four synthesised key findings: family‐centred care components identified in the dental attendance, aspects of interventions and settings that facilitated their acceptability, influence of oral health conversations and instructions on parents' behaviour, and barriers that persist. Confidence in the synthesised findings was rated as moderate–high. Parents mentioned aspects concerning the professionals, the impact of information on behavioural changes, the acceptability of dental interventions, expectations, criticisms and difficulties.

**Conclusion:**

The dentist's attitude is a crucial aspect of parents' experience and can influence the acceptability of interventions and the change in behaviours related to oral health.

**Summary**

- Why this paper is important to pediatric dentists
  ○ Knowing different aspects of parents' perceptions of their children's dental treatment is the first step in applying a family‐centred care approach.
  ○ Parents recognise when family‐centred care strategies are implemented, and communication during dental appointments significantly influences their satisfaction. Especially in pediatric dentistry, professionals need to communicate effectively with both adults and children, adapting language and terms to ensure clear and empathetic conveyance of oral health instructions.
  ○ Clinical practice guidelines often lack assessment of parents' views and values, which can be critical to decision‐making and adherence to treatment strategies; therefore, this document provides a valuable summary in this regard.

## 1 Introduction

A child's oral hygiene habits and oral health condition are directly influenced by the knowledge and attitudes of their family members [1]. Given that parents are responsible for establishing and maintaining an oral health care routine for their children, well‐informed parents can provide the child with support, guidance and assistance, which are essential to ensure the child's daily oral preventive care [2].

In dentistry, the first dental visit aims to provide families with accurate information and encourage parents to adopt the best dental care practices at home. Furthermore, the first dental visit seeks to prevent the onset of oral diseases and provide early intervention when necessary [3]. As children rely on parents for access to dental care [4] and despite widespread efforts to promote oral health practices globally, most children's first contact with a dentist may occur due to pain, poor oral function, appearance concerns or psychosocial impact [5]. Consequently, in addition to necessitating more invasive dental treatments, these conditions can lead to negative consequences that extend throughout life [5].

Understanding the experiences and perceptions of individuals involved in dental interventions for children can support clinical practice in paediatric dentistry, identify potential areas for improvement and contribute to the assessment of treatment acceptance. Furthermore, this information can be important for highlighting the need for changes in policies and practices, facilitating the development of health monitoring programmes and providing dental services aligned with users' needs and preferences [6].

Currently, to the best of our knowledge, no previous study has summarised the available evidence regarding parents' perspectives on dental treatment performed on their children through a qualitative approach. Thus, this systematic review aimed to identify, critically appraise and synthesise qualitative evidence on parents' experiences and perceptions of minimally invasive dentistry and invasive dental interventions for caries prevention and management in their children performed in different dental services.

## 2 Materials and Methods

The study protocol was registered in the International Prospective Register of Systematic Reviews (PROSPERO) database (protocol CRD42022382611). This systematic review was conducted following Joanna Briggs Institute (JBI) methodology for systematic reviews of qualitative evidence [7] and was reported according to the Preferred Reporting Items for Systematic Review and Meta‐Analysis (PRISMA) Statement [8].

### 2.1 Deviation From Protocol

Due to limited research in specific dental interventions, this systematic review emphasised preventive and management interventions for dental caries. A standardised tool was employed for data collection (refer to the data extraction section), with the inclusion of the ‘dental procedure’ item. Additionally, this review involves only parents, with a subsequent review planned to include children's findings.

### 2.2 Research Question

The PICo framework (Participants, Phenomenon of Interest and Context) was considered to formulate the following research question: ‘What are parents' experiences and perceptions of minimally invasive dentistry and invasive dental interventions for caries prevention and management in their children in different dental services?’

### 2.3 Inclusion Criteria

#### 2.3.1 Participants

Parents of children of both genders, from birth up to 12 years old.

#### 2.3.2 Phenomenon of Interest

Parents' perspectives on their experiences and perceptions regarding dental interventions for the prevention and management of dental caries in their children. Minimally invasive dentistry interventions were considered, following the classification by Banerjee et al. [9], which includes non‐invasive interventions (e.g., counselling sessions, oral health instructions, motivational interviews, dietary guidance, toothbrushing demonstrations and application of topical fluoride products), micro‐invasive interventions (e.g., dental sealants and infiltrants), minimally invasive interventions (e.g., restorative procedures with selective caries removal, including Atraumatic Restorative Treatment [ART]), and mixed‐method interventions (e.g., Hall Technique [HT] and non‐restorative cavity control). Additionally, invasive dental interventions were considered, which involve dental anaesthesia, complete caries removal and tooth preparation for the placement of Preformed Metal Crowns (PMC).

#### 2.3.3 Context

The context encompasses dental care services provided in private and public facilities, along with educational environments such as dental schools, primary schools and community centres in urban and rural areas.

#### 2.3.4 Types of Study

This review exclusively considered studies that used qualitative data collection methods (individual interviews or focus groups). Mixed‐methods studies were included when a comprehensive qualitative section was available for independent evaluation.

### 2.4 Exclusion Criteria

Studies were excluded if they presented combined voices of parents and children or lacked proper identification; included parents of children with cognitive impairments, syndromes or rare diseases, or those who experienced abuse, alcohol or drug addiction; used protective stabilisation, sedation and general anaesthesia; carried out the data collection based on photographs, videos or drawings; and focused exclusively on the perception of the dental professional or the dental room.

### 2.5 Search Strategy

A three‐step search strategy was considered. First, an initial limited search on MEDLINE (PubMed) was conducted in August 2022, followed by an analysis of the text words contained in the title, abstract and index terms used to describe the potentially included articles. The search strategy, including identified keywords and index terms, was adapted for each information source. A second search was performed on MEDLINE (PubMed), Scopus (Elsevier), Web of Science–All Databases (Clarivate Analytics), Embase (Ovid), APA PsycNet and on ProQuest Dissertations and Theses Global for grey literature search on October 14, 2022. The search strategy was updated in MEDLINE (PubMed), Scopus (Elsevier), Web of Science–All Databases (Clarivate Analytics) and Embase (Ovid) on April 12, 2024. The full search strategies for all databases are provided in Appendix S1. The third search involved manually screening the reference lists of the studies assessed for eligibility for further relevant studies that could fulfill the inclusion criteria. No language limits were applied. The authors are proficient in comprehending articles in both Portuguese and English, and when articles in other languages were encountered, an online platform (www.deepl.com) was utilised. No publication date or publication status restrictions were applied.

### 2.6 Study Selection and Assessment of Methodological Quality

Two independent reviewers (LD and FHS), and in duplicate, participated in all phases of the studies' screening, eligibility and methodological quality assessment. The studies identified were managed using Mendeley Desktop (Elsevier, London, UK) to identify and exclude duplicate references. The remaining references were uploaded on Rayyan (Rayyan Systems Inc., Cambridge, USA) for screening titles and abstracts. The full text of the included studies was assessed in detail. Full‐text studies that met the exclusion criteria were excluded, and reasons for their exclusion were provided.

Eligible studies were critically appraised using the standard JBI Critical Appraisal Checklist for Qualitative Research, which consists of 10 questions with response options of ‘yes’, ‘unclear’ and ‘no’ [7]. This instrument assesses research quality by evaluating the alignment between research methodology and objectives, data collection, analysis and interpretation of results. It also verifies the researcher's cultural or theoretical positioning, participant representation, ethical approval and conclusions driven by data. Answers with missing data were classified as ‘unclear’, while questions with absent data were classified as ‘no’. Any disagreement at any stage was resolved through discussion or by a third reviewer (AFM).

### 2.7 Data Extraction

Data were extracted by a reviewer (LD) and checked by another (FVDS), using the standardised JBI Data Extraction Tool [7], which included specific details about the populations, context, culture, geographical location, study methods and the phenomenon of interest relevant to the review objective. Information regarding dental interventions was also collected.

Findings accompanied by illustrations were extracted using the same tool [7]. A finding was considered a verbatim extract of the authors' analytical interpretation and an illustration referred to a direct quotation of the participants' voices. For each finding and its corresponding illustration, the reviewers assigned a level of credibility: unequivocal (U)—findings accompanied by an illustration that is beyond reasonable doubt and therefore not open to challenge; credible (C)—findings accompanied by an illustration lacking clear association with it and therefore open to challenge; or not supported (NS)—findings are not supported by the data. These decisions were made based on the reviewers' interpretation and disagreements were resolved through discussion. The studies' corresponding authors were contacted by email up to two times to request missing or additional data, when necessary. They were given a 4‐week period to provide the required information. Studies were excluded if the information was not provided within this timeframe.

A Microsoft Excel spreadsheet was created to organise the tool items (Microsoft Corporation, Redmond, USA).

### 2.8 Data Synthesis and Analysis

Qualitative research findings were pooled using JBI SUMARI with the meta‐aggregative approach [10]. This involved aggregation or synthesis of findings to develop sufficiently similar categories, with at least two findings per category. Subsequently, one or more synthesised findings of at least two categories were developed. In this study, the findings evaluated as U and C were grouped based on their similarity in meaning. The categories were created by a reviewer (LD). Finally, all the reviewers discussed the categories and created the synthesised findings and descriptions through a consensus process.

The final synthesised findings were graded according to the ConQual [11] to establish confidence in the output of qualitative research synthesis.

## 3 Results

### 3.1 Search Results

**FIGURE 1:**
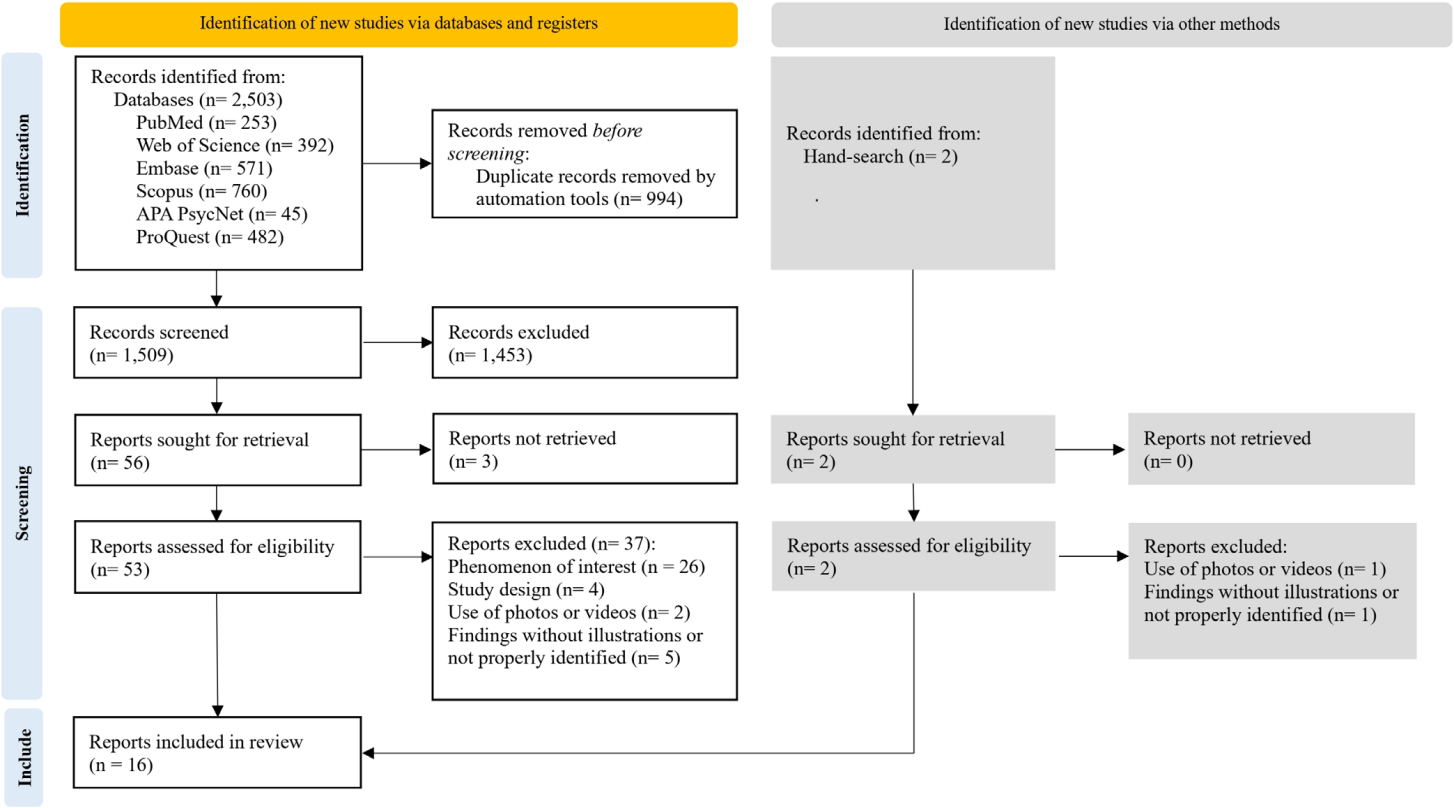
PRISMA flow diagram.

The PRISMA flowchart shows the study selection process (Figure 1). The systematic search identified 2503 publications. After duplicate removal, 1509 titles and abstracts were screened and 1453 studies were considered ineligible for not meeting inclusion criteria. At the full‐text review stage, three studies were not retrieved, and 53 studies were assessed and screened by two reviewers (LD and FHVS). Two studies were identified from the reference lists. Of the 55 studies assessed for eligibility, 39 were excluded based on the exclusion criteria. The list of all excluded studies and the reasons for exclusion are shown in Appendix S2.

### 3.2 Methodological Quality

Among the 16 included studies, question 1, which refers to the congruity between the stated philosophical perspective and the research methodology used in the studies, and question 6, which refers to a statement locating the researcher culturally or theoretically, showed the lowest scores. Two studies scored 10, four scored 9 and the remaining scored 8 (Table 1).

**TABLE 1:** Critical appraisal results for included studies using the JBI‐Qualitative Critical Appraisal Checklist.

Study	1	2	3	4	5	6	7	8	9	10	T
Arrow et al. (2021) [12]	U	Y	Y	Y	Y	N	Y	Y	Y	Y	8
Bhatti et al. (2021) [13]	U	Y	Y	Y	Y	Y	Y	Y	Y	Y	9
Bhatti et al. (2021) [14]	Y	Y	Y	Y	Y	N	Y	Y	Y	Y	9
Bhatti et al. (2022) [15]	Y	Y	Y	Y	Y	Y	Y	Y	Y	Y	10
Cashmore et al. (2011) [16]	U	Y	Y	Y	Y	N	Y	Y	Y	Y	8
Chai et al. (2022) [17]	U	Y	Y	Y	Y	N	Y	Y	Y	Y	8
Chestnutt et al. (2017) [18]	Y	Y	Y	Y	Y	N	Y	Y	Y	Y	9
El‐Yousfi et al. (2020) [19]	U	Y	Y	Y	Y	Y	Y	Y	Y	Y	9
Herval et al. (2019) [20]	U	Y	Y	Y	Y	N	Y	Y	Y	Y	8
Ihab et al. (2023) [21]	U	Y	Y	Y	Y	N	Y	Y	Y	Y	8
Kay et al. (2019) [22]	U	Y	Y	Y	Y	N	Y	Y	Y	Y	8
Kyoon‐Achan et al. (2021) [23]	U	Y	Y	Y	Y	N	Y	Y	Y	Y	8
Lee et al. (2022) [24]	Y	Y	Y	Y	Y	Y	Y	Y	Y	Y	10
Page et al. (2014) [25]	U	Y	Y	Y	Y	N	Y	Y	Y	Y	8
Schroth et al. (2016) [26]	U	Y	Y	Y	Y	N	Y	Y	Y	Y	8
Viswanath et al. (2021) [27]	U	Y	Y	Y	Y	N	Y	Y	Y	Y	8

*Note:* Q1: Is there congruity between the stated philosophical perspective and the research methodology?; Q2: Is there congruity between the research methodology and the research question or objectives?; Q3: Is there congruity between the research methodology and the methods used to collect data?; Q4: Is there congruity between the research methodology and the representation and analysis of data?; Q5: Is there congruence between the research methodology and the interpretation of results?; Q6: Is there a statement locating the researcher culturally or theoretically?; Q7: Is the influence of the researcher on the research, and vice versa, addressed?; Q8: Are participants, and their voices, adequately represented?; Q9: Is the research ethical according to current criteria or, for recent studies, is there evidence of ethical approval by an appropriate body?; Q10: Do the conclusions drawn in the research report flow from the analysis or interpretation of the data?

Abbreviations: N, no; Q, question; T, total; U, unclear; Y, yes.

### 3.3 Study Characteristics

Among the 16 included studies, publication dates ranged from 2011 to 2023. Thirteen studies [12, 13, 14, 15, 16, 17, 19, 20, 22, 23, 24, 25, 26] employed qualitative methods, while three [18, 21, 27] were mixed methods. The qualitative methodologies represented in the studies were action research [22, 24], descriptive research [13, 18, 20, 26], exploratory research [12, 14, 15, 16, 19, 21] and grounded theory [17, 23, 25, 27].

Interviews were conducted in all included studies. In three studies, the focus group method was used [12, 17, 26], while in the other studies, individual interviews were conducted. A total of 322 parents of children aged 0–11 years were interviewed. Most studies reported the gender of the parents, with a significant predominance of females.

The phenomenon of interest was reasonably homogeneous between the included studies and varied from perceptions, experiences, perspectives and acceptability of the dental interventions by parents.

All categories of interventions, both minimally invasive dentistry and invasive, were included in the studies analysed. However, some specific procedures, such as resin infiltrants and modified techniques, such as the Silver Modified Atraumatic Restorative Treatment (SMART) technique, were not included in the investigations. The use of non‐restorative cavity control has not been explicitly reported in any study. Further details on the included studies and the interventions performed in each of them can be found in Table 2.

**TABLE 2:** Characteristics of the primary qualitative and mixed‐method studies included.

Author (year) and country	Study design, methodology and method	Phenomenon of interest	Intervention and setting	Participants	Authors conclusion
Arrow et al. (2021) [12] Australia	Qualitative study, exploratory approach, focus groups at paediatric dental clinic	Parents' perspectives on the dental interventions	ART and Hall technique Public specialised dental service	8 parents (5 mothers and 3 fathers) of children with a mean age of 4.7 years	Parents of children with dental caries generally perceived the minimally invasive approach positively. The experience of timely and child‐centred care was of importance to parents and carers and positive impacts were reported where care had been received in such a manner
Bhatti et al. (2021) [13] England	Qualitative study, descriptive approach, Individual interviews (at parent's home) and focus groups (at children's centres, nurseries and primary schools)	Parent's experiences of dental consultations	Oral health conversations Unspecified setting	37 parents (gender not reported) of children aged 0–11 years	Many parents had little recollection of any preventive oral health conversations when visiting the dentist. Resistance to changing oral health behaviours and managing these conversations has identified the need for training and support around specific behaviour change techniques. In particular, how to have a personalised non‐judgemental two‐way conversation, rolling with resistance and understanding the wider context of those involved in the child's daily routine
Bhatti et al. (2021) [14] England	Qualitative study, grounded theory, semi structured interviews at participants' home	Parents' acceptability of the research process and the intervention	Oral health conversations with digital and paper‐based resources and toothbrushing instructions Public primary dental service	20 parents (gender not reported) of children aged 0–5 years	The intervention was acceptable to parents. In terms of affective attitude, parents valued the intervention resources and felt it integrated well within their family life and practice
Bhatti et al. (2022) [15] England	Qualitative study, exploratory research, semi‐structured interviews at participants' home	Parents' acceptability of the intervention	Oral health conversations with resources, including a website, videos, a toothbrushing demonstration and a leaflet with an oral health action plan Participants' home	17 parents (all mothers) of children aged 9–12 months	The intervention was acceptable to parents, but several contextual factors influenced the level of acceptability. These included establishing a relationship with the health visitors, the timing of the visit, family dynamics and the need for consistency and availability of support from other professionals
Cashmore et al. (2011) [16] Australia	Qualitative study, grounded theory, semi‐structured interviews at paediatric dental clinic	Parents' perceptions of the intervention	Counselling sessions with oral hygiene demonstrations, supportive resources (toothpaste and toothbrush, pamphlets and a brushing reward sheet) and fluoride applications (if tolerated by the child) Public specialised dental service.	12 parents (10 mothers and 2 fathers) of children aged under 5 years	Most parents believed they successfully improved their child's tooth‐brushing frequency and quality due, in part, to the parent‐supervised brushing demonstrations during counselling. Some even mentioned that this increased brushing lowered their child's dental pain, subsequently enhancing the child's quality of life
Chai et al. (2022) [17] Japan	Qualitative study, grounded theory, focus groups in kindergartens	Parents' perspective on the use of SDF	Oral health education seminar for parents and SDF applications on children's teeth Kindergartens	49 parents (44 mothers and 5 fathers) of children aged 4–5 years	The outreach dental service using SDF to treat early childhood caries in kindergarten is an acceptable strategy from their parents' point of view. The parents preferred caries arrest over aesthetics and accepted SDF therapy. However, some parents worried about SDF toxicity
Chestnutt et al. (2017) [18] Wales	Mixed‐method study, no specific philosophical framework mentioned, individual interviews by phone	Parents' acceptability of the interventions and setting	Pit and fissure sealant and fluoride varnish Primary schools	62 parents (49 parents of children aged 6–7 years in the 1st year; and 30 parents of children aged 8–9 years in the 3rd year. Of these, 17 parents participated in the follow‐up interview and 13 parents were included to increase the overall number of interviews) (gender not reported)	The acceptability of the treatments was very high. Parents reported trust in the school and convenience of access as important factors in using the service. The child‐friendly nature of the setting, the longer‐term and ongoing relationship of the service with the participating schools and the convenience of attending in school were all factors affecting the acceptability of the treatment setting for parents
El‐Yousfi et al. (2020) [19] Scotland and England	Qualitative study, Exploratory research, Semi‐structured interviews conducted on participant's home or another convenient location	Parents' acceptability of the interventions	Group 1: tooth brushing, dietary investigation, fissure sealants and fluoride varnish applied. Group 2: local anaesthetic, complete carious tissue removal, tooth preparation and placement of a filling or PMC. Group 3: selective caries removal and sealing of the tooth with an adhesive restorative material, no caries removal and sealing with a fissure sealant and PMC using the Hall Technique to restore the tooth. Groups 2 and 3 also received the Group 1 intervention Public primary dental service	13 parents (10 mothers and 3 fathers) of children aged 5–11 years	Parents found the strategies for the management of dental caries in primary teeth acceptable, with trust in the dental professional playing an important role
Herval et al. (2019) [20] Brazil	Qualitative study No specific philosophical framework mentioned, Semi‐structured interviews at health centre or mothers' home	Mothers' perceptions about the intervention	Health education actions Public primary dental service	12 parents (all mothers) of children under 1 year	Mothers perceive the educational actions to be necessary only for first‐time pregnant women and guidance from parents and maternity hospitals as important for childcare. Although the actions developed in Primary Health Care are not suitable to address the cultural context of mothers, there are multiple practices for oral health care being used and there is a lack of adherence to the guidelines provided by primary services
Ihab et al. (2023) [21] Egypt	Mixed‐method study, No specific philosophical framework mentioned, Semi‐structured interviews at health centre or mothers' home	Mother's acceptability of the intervention components	Motivational interviewing, oral health promotion messages and storytelling videos Dental school and mobile phones	16 parents (all mothers) of children between 2 and 5 years	The mothers expressed positive affective attitude toward the interventions and the three components were acceptable to the mothers. They felt the components served as ‘good reminders’ to brush their children's teeth
Kay et al. (2019) [22] England	Qualitative study, Action research, Semi‐structured interviews at participants' home	Parents' ethical, social and empirical acceptability of the intervention	Motivational interviewing. Participants' home	10 parents (gender not reported) of children between 21 days and 23 months	Establishing a trusting relationship with vulnerable families can motivate them to adopt oral health behaviours and seek dental services, making the intervention feasible and effective. Under these circumstances, families succeeded in embracing oral health practices and attending dental appointments
Kyoon‐Achan et al. (2021) [23] Canada	Qualitative study, Grounded theory, Semi structured interviews in the clinical environment and by phone	Parents' views on SDF	SDF and sodium fluoride varnish Community clinics	19 parents (14 mothers and 5 fathers) of children under 6 years	Most parents were accepting of SDF as a non‐surgical treatment to arrest caries and minimise dentinal sensitivity secondary to caries, although some expressed concern about the black staining in anterior teeth
Lee et al. (2022) [24] Canada	Qualitative study, Community‐based participatory research, Semi structured in‐person interviews at a local film studio	Indigenous parents' perspectives on oral health care for their children	Oral health anticipatory guidance, motivational interviewing, SDF and sodium fluoride varnish Community clinics	6 parents (4 mothers and 1 father) of children aged under 6 years	The digital storytelling method facilitated interactions and engaged Indigenous parents in creating a digital representation of oral health in general and their experiences caring for children with early childhood caries
Page et al. (2014) [25] New Zealand	Qualitative study, Grounded theory, Individual in‐depth interviews by telephone	Parents' perceptions of the intervention	Stainless steel crowns placed using the Hall Technique Community clinics	10 parents (7 mothers and 3 fathers) of children aged 6–7 years	The findings support an overall positive reaction to the Hall Technique from the parents. Individual feedback from some parents suggests that specific aspects of the treatment were less than ideal. Perhaps more emphasis should be placed on informing parents of possible complications and alterations to the treatment plan and investigating how clinicians can act to ensure that these complications do not arise
Schroth et al. (2016) [26] Canada	Qualitative study, Descriptive research, Focus group conducted at or near parenting programme sites	Parents' perspectives on a free dental first visit program	Oral health instructions and the child's dental screening Dental offices	21 parents (19 mothers and 2 fathers) of children aged 1–5 years	Many participants liked the programme and believed that it should continue. Parents would benefit from further education and encouragement to seek oral care for their child by age one
Viswanath et al. (2021) [27] India	Mixed‐method study, Grounded theory, Semi‐structured interview at the participant's home/workplace	Parents' views on the intervention	Child's first dental visit Dental offices and primary schools	10 parents (6 mothers and 4 fathers) of children under 6 years	Lack of interdisciplinary practices among different health care professionals and lack of awareness had a significant impact on the child's first dental visit. Overcoming these barriers would be beneficial for all children belonging to various cultural and socioeconomic backgrounds from different parts of the world

### 3.4 Review Findings

**FIGURE 2:**
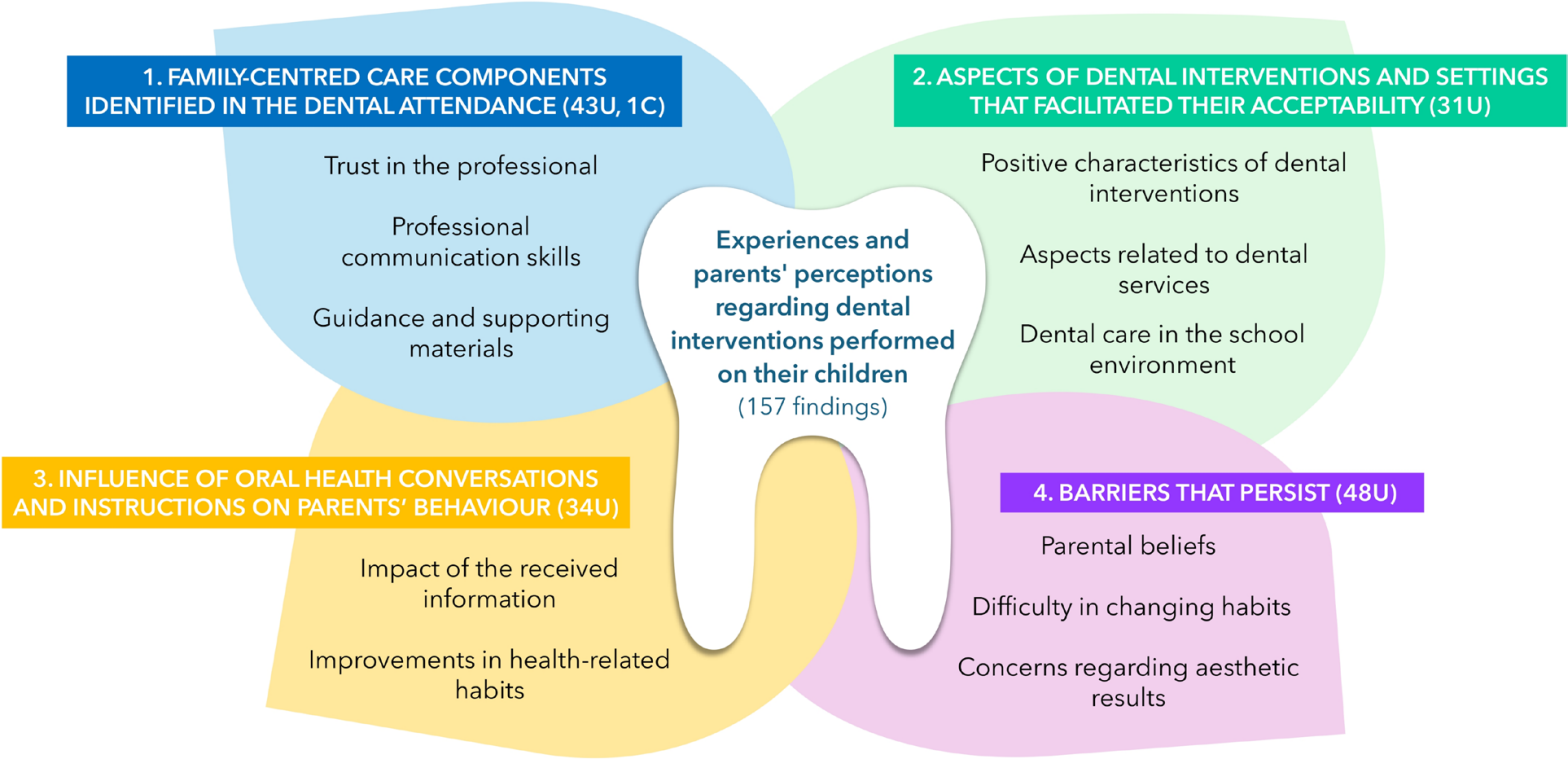
Themes of the 4 synthesised findings, involving 157 findings, and the 12 categories that comprise them, regarding experiences and parents' perceptions of dental interventions performed on their children.

Among the 16 included studies, 193 findings were identified. Of these, 156 findings were rated as unequivocal, 1 as credible and 36 as not supported. The 157 findings (U and C) were aggregated into 11 categories based on the similarity of meaning, concepts or ideas voiced within the illustrations. This process generated four synthesised findings: (i) family‐centred care components identified in the dental attendance; (ii) aspects of interventions and settings that facilitated their acceptability; (iii) influence of oral health conversations and instructions on parents' behaviour and (iv) barriers that persist. Figure 2 presents the themes of the four synthesised findings and the 12 categories that comprise them. Appendix S3 presents a complete list of the categories with their associated findings and illustrations.

#### 3.4.1 Synthesised Finding 1: Family‐Centred Care Components Identified in the Dental Attendance

Three interrelated categories, comprising 43 U and 1C findings, were integrated into synthesised finding 1. This synthesised finding describes some aspects that parents consider important in dental care, which help increase their confidence and well‐being in the dental environment. These aspects include the professional's attitude and conduct, which foster the building of trust between them and the family; the professional's communication skills, which ensure clear interactions, including with the child; and the quality of the guidance and materials provided to families.

##### 3.4.1.1 Category 1: Trust in the Professional

Although this review does not focus on perceptions regarding the professionals who perform the interventions, many parents mentioned that trusting the professionals, such as dentists or health visitors, significantly influences their choices and the acceptability of interventions [15, 19, 23]. In home‐based counselling sessions, parents emphasised the importance of establishing a strong relationship with the health visitor because this connection made the visit more personalised due to the health visitor's prior knowledge of the child's health [15]. Similarly, in clinical settings, parents believe that receiving ongoing care from the same dentist helps build trust [16, 19]. They feel that having a professional, rather than family members, providing guidance has a more significant impact and promotes behaviour change in the child, encouraging them to be involved in their oral health care [13, 16, 17].

In invasive dental interventions, mainly because this treatment requires local anaesthesia, the professional's approach was considered crucial for building trust with the family and the child, which led one father to describe the conduct as fantastic [19]. On the other hand, parents expressed concerns about the involvement of general dentists, who may be less familiar with paediatric treatments, often opting to extract primary teeth instead of opting for interventions to preserve them [27]. The lack of interdisciplinary collaboration among other professionals involved in the child's care and paediatric dentists was identified as a factor impacting paediatric dental care, while the opportunity to be referred to a child‐friendly dentist was considered a valuable aspect [22, 27].

##### 3.4.1.2 Category 2: Professional Communication Skills

Parents expressed a preference for a non‐confrontational, patient‐centred conversation style, indicating that they did not want to be ‘lectured’ about their child's oral health or feel judged [16]. In some cases, this preference could be attributed to their feelings of guilt or embarrassment regarding the condition of their child's teeth [16]. Some parents reported that preventive interventions can facilitate a friendly conversation without imposing, enabling a two‐way dialogue [14, 18]. Parents also appreciate the empathy demonstrated by the dentist toward their children, valuing when dentists listen to them, ask questions about activities like school, and respond with kindness [12, 19]. They acknowledged the skills of the dentist and the service team in making the child feel comfortable, particularly in alleviating dental fear and anxiety, especially in the case of uncooperative children [12, 19].

##### 3.4.1.3 Category 3: Oral Health Guidance and Supporting Materials

Parents emphasised that practical guidance enables them to identify problematic toothbrushing techniques and enhance their brushing methods [12, 14, 19]. When only oral information was transmitted, there was a higher probability that they would forget it [13]. In the case of leaflets, parents indicated a preference for these materials to be clear and objective. They appreciate when professionals not only provide the material but also explain its content, allowing parents the opportunity to ask questions and better understand the information [14]. These materials were perceived as a means to disseminate information to other family members and friends, serving as a way to promote positive behaviours at home [14, 15]. Regarding materials shared through online resources, mothers reported that these served as reminders to brush their children's teeth and motivated them to take action [21]. They found the storytelling videos relatable and reflective of real‐life experiences. Mothers viewed the stories as an effective way to encourage other mothers to care for their children's teeth [21].

#### 3.4.2 Synthesised Finding 2: Aspects of Dental Interventions and Settings That Facilitated Their Acceptability

Three related categories encompassing 31 U findings were integrated into synthesised finding 2. This synthesised finding summarises the aspects highlighted by parents as facilitators for dental care. This includes the lower degree of invasiveness of minimally invasive dentistry interventions, attributes of dental services that facilitated access, such as free services and proximity to the place of residence, as well as the benefits of interventions carried out in a school environment.

##### 3.4.2.1 Category 1: Positive Characteristics of Minimally Invasive Dentistry

Parents believe that minimally invasive dentistry interventions helped reduce their children's fear [19] and were beneficial in both preventing and treating dental caries [17]. Regarding Silver Diamine Fluoride (SDF) treatment, parents emphasised its painless nature, avoidance of dental drills and restorations, reduced sensitivity, and its effectiveness in halting the progression of their child's caries lesions [23]. Although parents noted changes in tooth colour, they considered all the previously mentioned aspects to be more important, emphasising the significance of oral health over dental aesthetics [17, 24]. Furthermore, parents indicate that they have recommended the SDF technique to other parents because they consider the treatment helpful [23, 24].

Concerning dental sealants, ART and the HT, parents emphasised the positive aspect of avoiding the use of drills, injections [19, 25] and dental extractions [12]. This can be attributed to parents' desire to prevent their negative experiences from being repeated with their children [19]. Parents expressed that having their child in the dental chair, feeling comfortable and confident about going to the dentist, made them feel better, as they knew their child received the necessary treatment without having to be completely anaesthetised [12]. Specifically, concerning the HT, parents emphasised that aesthetic concerns were not a priority; instead, the avoidance of injections and time‐saving were considered more important factors [19, 25].

##### 3.4.2.2 Category 2: Aspects Related to Dental Services

Parents highlighted aspects related to the service where the dental interventions were carried out. Free interventions were seen as an important way to access dental care, whether early treatment or specialised care [12, 26]. In Schrot et al. [26], where a first dental visit programme for children under 3 years of age was integrated into a government‐sponsored initiative in Canada, parents reported that although not all dental needs were addressed during the first appointment, the programme proved to be an effective approach that benefits many people who may not have had this opportunity before. Furthermore, parents appreciate interventions conducted in their communities, eliminating the need for long‐distance travel to centres or even to other cities [12]. In studies where mothers had to travel to receive the intervention, they reported that it was difficult for them, and they tried to find solutions such as going to the interventions on days off from work during the month [21]. They also reported that online interventions required no effort on their part, as they always have their cell phones nearby and that online information could reach more people. The mothers emphasised that a significant portion of the population currently has access to the internet at home and that there would be no additional costs associated with the preventive interventions provided online [21].

##### 3.4.2.3 Category 3: Dental Care in the School Environment

Numerous parents expressed the belief that schools and kindergartens were favourable environments for dental check‐ups and for teaching children the importance of dental visits [17, 18, 27]. Parents stated that their children behaved better, made less fuss or were less nervous in these settings, which could alleviate the child's dental fear and anxiety, preparing them for dental examinations in the future [17]. In Chesnutt et al. [18], one parent stated that their child's participation in the study was influenced partially by the fact that the intervention would take place at school, reflecting the trust parents place in the school environment.

#### 3.4.3 Synthesised Finding 3: Influence of Oral Health Conversations and Instructions on Parents' Behaviour

Two related categories encompassing 34 U findings were integrated into synthesised finding 3. This synthesised finding describes how the information provided by professionals impacted parents' oral health care practices for their children, as well as the improvements observed in their children's care routines and overall well‐being.

##### 3.4.3.1 Category 1: Impact of the Received Information

Guidance on the optimal timing to start brushing, as well as the selection of toothbrushes and toothpaste, significantly contributed to strengthening parents' confidence in adopting appropriate oral care practices [15]. They expressed that understanding the potential harm of dental caries, such as the negative impact on their children's eating and concentration at school, had a strong motivational effect on implementing oral hygiene practices at home [21]. And, based on the knowledge gained, they felt committed to avoiding repeating past mistakes [17, 24].

Even though some parents stated that the instructions they received were not new information, they recognised that this information helped them realise that they were already adopting the correct practices at home [15, 21]. Meaningfully, parents actively retained and applied the educational messages received in dental clinics, both for their children and themselves [18]. They expressed a keen interest in maintaining ongoing vigilance over their children's oral health status. Some parents focused on monitoring the progression of tooth decay [16], while others emphasised the importance of regular dental visits, even in the absence of tooth decay [17]. Additionally, parents noted that regular assessments help maintain children's motivation to brush their teeth, as they strive to achieve a better evaluation with each visit [17].

##### 3.4.3.2 Category 2: Improvements in Health‐Related Behaviours

Parents reported an improvement in their children's behaviours and, consequently, in their daily routines following the dental interventions. These improvements were observed in their children's toothbrushing habits [12, 16, 17, 19, 22, 24], reduction in dental pain [12, 16, 17, 19], sleep patterns [12, 16], absence of feeding difficulties [16, 19], weight gain [12] and reduction in sugar consumption [19]. Furthermore, some parents acknowledged that with the improvement in children's oral health, their own sense of well‐being increased, reducing stress and feelings of distress [12, 16]. To achieve these results, some parents explained that establishing a daily routine that connects toothbrushing with other household activities (such as bathing and dinner) was crucial [16, 22]. Parents also recognised the effectiveness of strategies that enhance children's engagement in practising oral hygiene at home, simplifying routines and promoting greater acceptance of oral health practices. They emphasised the positive impact of oral hygiene products designed for children, such as electric toothbrushes, flavoured toothpaste and items featuring popular TV characters [13, 14, 16, 18]. They also recognised a ‘brushing rewards sheet’ as an effective method to encourage positive behaviours and motivate children to brush their teeth [16]. Moreover, children with older siblings often perceive them as role models and seek to imitate their behaviour, facilitating the process [15, 16].

#### 3.4.4 Synthesised Finding 4: Barriers That Persist

Three related categories encompassing 48 U findings were integrated into synthesised finding 4. This synthesised finding highlights parental beliefs that may impede the prevention and management of dental caries through less invasive strategies and can negatively affect a child's behavioural adaptation to the dental environment. These beliefs are reflected in their expectations and the ongoing persistence of their current habits.

##### 3.4.4.1 Category 1: Parental Beliefs

Some parents expressed that they were unaware that dental caries could be prevented [16, 27]. Others did not recognise the importance of attending preventive check‐ups and dental treatments for primary teeth, as these teeth would eventually fall out [17, 19, 27]. Consequently, a parent who witnessed an invasive treatment involving anaesthesia and perceived it negatively questioned the necessity of subjecting the child to such an unpleasant experience [19].

Additionally, some parents believed that they could assess their children's oral health at home, and if they did not notice any obvious problems, they saw no need to visit a dentist [17]. This belief was reinforced by their experiences with older children, as well as their trust in family advice [20] and their ability to research online [27]. Furthermore, when their children experienced dental pain, some parents tried using home remedies and over‐the‐counter medications before seeking professional treatment, which made the child's first dental experience unpleasant [27].

On the other hand, parents whose children participated in a free first‐visit programme in Canada reported that they expected more than just a screening and felt disappointed, stating that the opportunity of the free appointment was not fully utilised [26]. In the same study, one participant disagreed with the recommended age for a child's first dental visit and pointed out that it would be important for the child to have more than one tooth to make the most of the free appointment [26]. Other parents engaged in preventive programmes expressed concerns about the potential for further deterioration of the primary teeth and a possible impact on the child's permanent successor tooth due to the use of less invasive strategies [19]. One parent stated that if their children experienced pain, they expected a more radical approach to be taken, such as restoration or even dental extraction [19].

In the parents' discourse, it was also possible to identify their own dental fear and anxiety due to their previous experiences [19, 25, 26]. They expressed concern about potential pain that the child might feel during the procedure or the child's resistance to the drill, anticipating that the child would not like it and would have difficulties returning for a follow‐up appointment [19]. They also highlighted that, in previous situations, when the child had contact with the drill, they did not like it, and therefore, it would be difficult to use it again [25]. Also, a parent stated that they remembered the pain of receiving anaesthesia and preferred that their children did not undergo that intervention [19].

##### 3.4.4.2 Category 2: Difficulty in Changing Habits

Some parents faced difficulties in reducing their children's sugar consumption due to the children's misbehaviour when denied sweets [16]. By the way, they recognised that adults had the same difficulty in making good choices, even when they knew what the healthiest food options were [13]. In this aspect, parents see schools as a reinforcement in the dietary care of children. They stated that, at home, sugar consumption can be restricted, but it will be available in school snacks. And, although children have other dessert options, like fruits, they tend to choose the sugary options [13]. Parents of younger children also encountered challenges when attempting to conduct supervised toothbrushing at home, as they may exhibit more resistance to brushing [15]. According to the parents, forcing the process could lead to a negative experience for the children [15]. Additionally, the challenges were compounded by the demands of a busy family life with competing priorities [15]. A mother of an older child stated that, over the years, her son became lazy about brushing his teeth and that, if he is home only with his dad, he won't brush. This expresses how some mothers felt their partners were not as supportive in maintaining optimal oral health for their children [13]. Devices that could assist with brushing, such as mobile applications, were not well‐received by some parents, as they were considered burdensome [14]. Additionally, the use of an electronic device in the bathroom disrupted the routines that parents had established with their children [14].

##### 3.4.4.3 Category 3: Concerns Regarding Aesthetic Results

Some parents describe that their acceptance of SDF treatment was associated with the exfoliation of deciduous teeth [17] and the application of the technique on posterior teeth, which would not be visible during the child's speech or smile [23]. Although parents mentioned being informed about potential tooth discoloration changes, they expressed discomfort with the result. Both for SDF treatment and for PMC, parents reported concerns that other adults might assume they took no action regarding the caries lesions, and the lesions might appear more severe [17, 19, 24]. Parents believe that children accept a non‐aesthetic treatment due to their young age, but as the years pass and they start going to school, this can become a problem [19, 23].

### 3.5 Confidence in the Findings

The synthesised findings 2, 3 and 4 had no dependability and credibility levels downgraded, with confidence levels classified as high. Only synthesised finding 1 was downgraded one level due to a mixture of unequivocal and equivocal findings, with confidence levels classified as moderate (Table 3).

**TABLE 3:** Summary of findings (ConQual Score).

Synthesised finding	Type of research	Dependability	Credibility	ConQual score	Comments
Synthesised finding 1: Family‐centred care components identified in the dental attendance	Qualitative and Mixed‐method study	No change	Downgrade 1 level (−1)	High	Dependability: The classification was not changed because all studies presented 4–5 ‘yes’ answers in the dependability evaluation Credibility: Downgraded one level due to a mix of unequivocal and equivocal findings (43U, 1C)
Synthesised finding 2: Aspects of dental interventions and settings that facilitated their acceptability	Qualitative and Mixed‐method study	No change	No change	High	Dependability: The classification was not changed because all studies presented 4–5 yes answers Credibility: The synthesised finding contains only unequivocal findings (31U)
Synthesised finding 3: Influence of oral health conversations and instructions on parents' behaviour	Qualitative and Mixed‐method study	No change	No change	High	Dependability: The classification was not changed because all studies presented 4–5 ‘yes’ answers in the dependability evaluation Credibility: The synthesised finding contains only unequivocal findings (34U)
Synthesised finding 4: Barriers that persist.	Qualitative and Mixed‐method study	No change	No change	High	Dependability: The classification was not changed because all studies presented 4–5 ‘yes’ answers in the dependability evaluation Credibility: The synthesised finding contains only unequivocal findings (48U)

*Note:* Systematic review title: Experiences and parents' perceptions regarding dental interventions performed on their children: a qualitative systematic review. Population: Parents of children of both genders, from birth up to 12 years old. Phenomenon of interest: Parents' perspective describing their experiences and perceptions about minimally invasive dentistry interventions and invasive dental interventions for caries prevention and management in their children. Context: Dental care services provided in both private and public facilities, along with educational environments such as dental schools, primary schools and community centres, in both urban and rural areas.

## 4 Discussion

The included studies in this qualitative systematic review showed different viewpoints of parents around the world regarding dental interventions. However, a key point identified in the speech of parents is the relationship between them, the children and the professional. Parents were able to recognise when patient‐centred care strategies were implemented, and communication during dental appointments was a significant factor in their satisfaction [2]. Especially in paediatric dentistry, professionals need to communicate with both adults and children. Enhancing clinicians' communication skills is one approach to creating a sense of warmth, increasing the acceptability of dental interventions and fostering treatment cooperation among children [2]. Therefore, it is important for professionals to assess the family's level of knowledge and adapt the language and terms used to ensure that oral health instructions are conveyed clearly [28]. Traditional oral health education, which does not take into account the individual's interpersonal context and subjectivities, often does not result in positive clinical outcomes [29]. In this sense, strategies such as motivational interviewing are being utilised in paediatric dentistry. Through a collaborative communication style, this technique allows families to express their doubts and share their knowledge. Conversely, professionals can guide health behaviour changes based on the individual's values and resources [30].

As facilitators of access to dental treatments, parents mentioned the reduced costs [12, 26], the proximity of the service location [12] and the delivery of interventions in schools and kindergartens [17, 18, 27]. Although this review did not aim to capture the sociodemographic characteristics of the participants, it is known that families with more children, low income or residing in deprived areas may face increased stress due to multiple demands associated with health [4]. Considering variations in the public healthcare system across different countries or supplementary insurance options, the cost of treatment [27, 31] and the distance to facilities [3] could be determining factors in the decision‐making process.

Parents who participated in counselling sessions reported observing improvement in habits at home, which consequently improved the family routine, the child's well‐being and their well‐being. Preventing oral diseases in children requires the constant involvement of parents in establishing or improving a child's oral health and continuous effort until the child develops self‐care skills [2]. By the way, it is not uncommon to observe parents who believe they already possess the necessary knowledge and, often, engage in incorrect oral hygiene practices [15, 17, 20].

Misinformation continues to perpetuate numerous barriers to dental care, as it generates expectations that adversely affect the reception of information and the decision‐making process regarding dental treatment for children [4]. The use of the internet by families to seek information about dental treatments should be recognised as a way to facilitate their access to knowledge [31]. However, it is currently estimated that most online information about oral health has inadequate content, lacks verification and is developed by professionals with specific interests other than oral health promotion [32]. This highlights the low number of materials produced by dental professionals with scientific expertise in specific topics, who should be aware of their significant responsibility to promote quality oral health information committed to good health practices [32]. Rigorously developed clinical practice guidelines published on open platforms using accessible communication could potentially fill this gap, assisting dentists, oral health policy makers and the general population.

Regarding minimally invasive dentistry interventions, the results do not allow for generalising the acceptability of a strategy or dismissing the possibility of a treatment. Thus, within the same group of parents, perception can vary from individual to individual, depending on what each one considers most suitable [33]. For example, regarding the SDF and HT interventions, some highlighted positive aspects, while others expressed a preference for treatments that make the tooth look more natural. Research in the field shows a tendency for parents to prefer more aesthetic treatments [23, 33], but it is also crucial that the context in which the treatment is conducted, such as access to material resources, stage of primary tooth resorption, child's cooperation and the operator's skills be considered for the indication of a strategy.

On the other hand, more negative views were observed regarding invasive treatments involving local anaesthesia and dental drills. Some parents consider these procedures ‘traditional’ due to a lack of awareness of alternatives [19], while others, influenced by past negative experiences [4], prefer to avoid them [23, 33]. These procedures often induce anxiety in both children and their parents and, at times, are the only available option. Although no studies in this review reported the use of rubber dams, they may also be linked to these negative feelings. As noted earlier, future efforts should focus on improving clinicians' communication skills to better prepare parents and children for more invasive procedures, such as those involving local anaesthesia. Providing clear explanations about treatment necessity, expected outcomes and coping strategies may improve treatment acceptance and reduce procedural anxiety. Pre‐appointment resources, such as informational leaflets, videos or role‐playing exercises, could further support parents in preparing their child effectively. By equipping parents with the right tools and knowledge, clinicians can help foster a more positive treatment experience, reducing anxiety and improving overall treatment acceptance. In addition, more studies are needed to evaluate parents' qualitative perspectives on invasive procedures to understand why some parents view these procedures negatively and, conversely, what motivates those who accept and support them to approach them positively.

The limitations of this review must also be addressed. Although all studies employed qualitative methods for data collection, the sampling methods varied between studies. The participants were parents who accompanied their children during a specific treatment, participated in single‐arm intervention studies or randomised clinical trials. This means that parents did not have the option to choose the interventions, only to decide whether to participate in the dental treatments. Also, regardless of the interventions performed, both minimally invasive and invasive, the studies did not provide information on explaining the risks of treatment failure and the need for reinterventions to parents, nor how these risks could influence the children's behavioural habits at home while waiting for treatment or after it. In just one study on non‐invasive interventions [16] planned to prevent further deterioration of children's teeth awaiting dental treatment under general anaesthesia, an extreme situation, parents reported changes in home habits. Future studies may be useful in investigating whether, given the possibility of choice, parents would prefer different alternatives and whether exposure to detailed pre‐treatment information leads to improved preventive care at home.

In studies reporting respondent gender, mothers predominantly participated, which is expected given their greater involvement in various aspects of daily childcare and more frequent presence during healthcare activities [34]. This gender imbalance may skew the findings toward maternal viewpoints, as mothers and fathers often have distinct perspectives on health interventions for their children. Consequently, a significant portion of the perceptions gathered—and thus the findings—reflect a maternal perspective. Globally, women perform most of the unpaid care work, dedicating 55% of their non‐leisure time to domestic tasks and childcare, compared to 19% for men. Although gender norms are changing, this process is slow in some countries and varies significantly by geographic region, cultural beliefs and the age of parents, for example [35]. Future studies examining the differences in perceptions between mothers and fathers regarding child oral health interventions would provide insights into how their unique experiences and roles influence caregiving decisions.

Also, the included studies were conducted in different countries with distinct geographical and cultural contexts, and these factors can shape individuals' experiences and perceptions regarding dental interventions. The distribution of dental caries in children worldwide is not homogeneous, with the highest prevalence seen in those living in socioeconomically disadvantaged countries. Consequently, inequality, poverty, educational levels, living in urban or rural areas, sugar consumption, access to fluoride, the presence of programmes, coverage and the different oral healthcare systems can influence the value placed on a dental service or treatment and affect the significance of care for children's teeth. For instance, in countries with limited access to public health services, parents may prioritise immediate health concerns over preventive measures, impacting their perceptions of oral health interventions [36, 37, 38].

It is relevant to highlight that only two of the 16 studies reported adhering to a specific reporting guideline for qualitative research. Adherence to standardised reporting instruments can enhance methodological description, as well as identify factors that potentially impact the confidence in findings, such as the theoretical and cultural influence of the researcher on data collection and analysis, thus improving the methodological quality of a study. Studies using qualitative methods can provide more detailed insights and are gaining increasing visibility in dentistry. The effective use of this research methodology has significant potential to enhance the understanding of the expectations and experiences of individuals involved in dental interventions, contributing to improvements in healthcare.

In conclusion, this review summarised several aspects regarding children's dental care that were considered important from the parents' perspective. It can be highlighted that the dentist's attitude plays a relevant role in promoting oral health since it can influence health behaviours through information that parents consider useful and through an empathetic relationship. In addition, negative beliefs and experiences should be openly discussed so that barriers can be reduced. Professionals must develop their communication skills to convey health information and guidance on dental interventions to the family, thereby establishing a trusting relationship that encourages behavioural change toward health.

## Author Contributions

L.D.: conceptualization, methodology, data collection, data analyses, assessment of methodological quality, prepare figures and tables, writing – original draft preparation. A.F.M.: conceptualization, methodology, data analysis, writing – reviewing and editing. T.K.T.: conceptualization, methodology, data analyses, writing – reviewing and editing. T.T.M.: conceptualization, methodology, data analysis, writing – reviewing and editing. F.H.V.S.: conceptualization, methodology, data collection, data analyses, assessment of methodological quality, writing – reviewing and editing. All authors reviewed and approved the final manuscript.

## Disclosure

PROSPERO Registration: protocol CRD42022382611.

## Ethics Statement

The authors have nothing to report.

## Consent

The authors have nothing to report.

## Conflicts of Interest

The authors declare no conflicts of interest.

## Supporting information


Appendices S1–S3

## Data Availability

Data is provided within the manuscript and Supporting Information files.
