# Supplementary material for: Experiences and Parents' Perceptions Regarding Dental Interventions Performed on Their Children: A Qualitative Systematic Review
Source: Int J Paediatr Dent. 2025 May 7;35(6):1029–45. doi: 10.1111/ipd.13318 (PMC12580900; doi:10.1111/ipd.13318)
Supplement: Supplementary file 1 — Appendices S1–S3 [file IPD-35-1029-s001.docx]

**Supporting information**

**Appendix S1:** Search strategy for all databases

**MEDLINE (PubMed)**Data searched: October 14, 2022 (updated on April 12, 2024)
Results: 253

| **Search** | **Search parameters** | **Results Oct 14, 2022** | **Results Apr 12, 2024** |
| --- | --- | --- | --- |
| 1 | “Child”[Mesh] OR “children”[tiab] OR “Preschool”[tiab] OR “Infant”[tiab] OR “Toddler”[tiab] OR “minor”[tiab] | 2,833,258 | 157,908 |
| 2 | “Perception”[Mesh] OR “Acceptability”[tiab] OR “Expectations”[tiab] OR “Preferences”[tiab] OR “Opinions”[tiab] OR "Experiences"[tiab] OR “Perspectives”[tiab] OR "Parents views"[tiab] | 1,065,628 | 109,102 |
| 3 | “Dental Care for children”[Mesh] OR “Dental Health Services”[tiab] OR “Oral health”[tiab] OR "Dental Care "[tiab] OR "Pediatric Dentistry"[tiab] | 47,800 | 5,861 |
| 4 | "Qualitative research"[Mesh] OR "Qualitative study"[tiab] OR "Qualitative exploration"[tiab] OR “Phenomenology” [tiab] OR “Grounded theory” [tiab] OR “Ethnography” [tiab] OR “Action research” [tiab] OR “Discourse analysis” [tiab] OR “Focus groups”[tiab] OR Interviews[tiab] OR "Mixed-method approach"[tiab] | 348,619 | 48,709 |
| 5 | S1 AND S2 AND S3 AND S4 | 222 | 31 |

**Web of Science (Clarivate Analytics)**Data searched: October 14, 2022 (updated on April 12, 2024)
Results retrieved: 392

| **Search** | **Search parameters** | **Results Oct 14, 2022** | **Results Apr 12, 2024** |
| --- | --- | --- | --- |
| 1 | TS=“Child” OR “children” OR “Preschool” OR “Infant” OR “Toddler” OR “minor” | 2,395,194 | 194,142 |
| 2 | TS=“Perception” OR “Acceptability” OR “Expectations” OR “Preferences” OR “Opinions” OR "Experiences" OR “Perspectives” OR "Parents views" | 1,816,110 | 236,614 |
| 3 | TS=“Dental Care for children” OR “Dental Health Services” OR “Oral health” OR "Dental Care" OR "Pediatric Dentistry" | 39,418 | 5,643 |
| 4 | TS="Qualitative research" OR "Qualitative study" OR "Qualitative exploration" OR “Phenomenology” OR “Grounded theory” OR “ethnographic” OR “Action research” OR “Discourse analysis” OR “Focus groups” OR Interviews OR "Mixed-method approach" | 863,918 | 112,953 |
| 5 | S1 AND S2 AND S3 AND S4 | 347 | 45 |

**Embase (Elsevier)**
Data searched: October 14, 2022 (updated on April 12, 2024)
Results retrieved: 571

| **Search** | **Search parameters** | **Results Oct 14, 2022** | **Results Apr 12, 2024** |
| --- | --- | --- | --- |
| 1 | 'child'/exp OR 'child' OR 'children'/exp OR 'children' OR 'preschool'/exp OR 'preschool' OR 'infant'/exp OR 'infant' OR 'toddler'/exp OR 'toddler' OR 'minor'/exp OR 'minor' | 4,761,653 | 446,074 |
| 2 | 'perception' OR 'acceptability' OR 'expectations' OR 'preferences' OR 'opinions' OR 'experiences' OR 'perspectives' OR 'parents views' | 1,210,631 | 170,049 |
| 3 | 'dental care for children' OR 'dental health services' OR 'oral health' OR 'dental care' OR 'pediatric dentistry' | 91,710 | 14,414 |
| 4 | 'qualitative research'/exp OR 'qualitative research' OR 'qualitative study'/exp OR 'qualitative study' OR 'qualitative exploration' OR 'phenomenology'/exp OR 'phenomenology' OR 'grounded theory'/exp OR 'grounded theory' OR 'ethnographic' OR 'action research'/exp OR 'action research' OR 'discourse analysis'/exp OR 'discourse analysis' OR 'focus groups'/exp OR 'focus groups' OR 'interviews'/exp OR interviews OR 'mixed-method approach' | 2,922,635 | 602,676 |
| 5 | (S1) AND (S2) AND (S3) AND (S4) | 470 | 101 |

**Scopus**Data searched: October 14, 2022 (updated on April 12, 2024)
Results retrieved: 760

| **Search** | **Search parameters** | **Results Oct 14, 2022** | **Results Apr 12, 2024** |
| --- | --- | --- | --- |
| 1 | TITLE-ABS-KEY ("Child" OR "children" OR "Preschool" OR "Infant" OR "Toddler" OR "minor") | 4,593,371 | 465,519 |
| 2 | TITLE-ABS-KEY ("Perception" OR "Acceptability" OR "Expectations" OR "Preferences" OR "Opinions" OR "Experiences" OR "Perspectives" OR "Parents views") | 5,661,556 | 1,033,892 |
| 3 | TITLE-ABS-KEY ("Dental Care for children" OR "Dental Health Services" OR "Oral health" OR "Dental Care" OR "Pediatric Dentistry") | 115,445 | 11,354 |
| 4 | TITLE-ABS-KEY ("Qualitative research" OR "Qualitative study" OR "Qualitative exploration" OR "Phenomenology" OR "Grounded theory" OR "ethnographic" OR "Action research" OR "Discourse analysis" OR "Focus groups" OR interviews OR "Mixed-method approach") | 1,193,474 | 259,930 |
| 5 | S1 AND S2 AND S3 AND S4 | 629 | 131 |

**APA PsycNet (EBSCO)**Data searched: October 14, 2022
Results retrieved: 45

| **Search** | **Search parameters** | **Results Oct 14, 2022** |
| --- | --- | --- |
| 1 | (“Child” OR “children” OR “Preschool” OR “Infant” OR “Toddler” OR “minor”) | 1,099,430 |
| 2 | (“Perception” OR “Acceptability” OR “Expectations” OR “Preferences” OR “Opinions” OR "Experiences" OR “Perspectives” OR "Parents views") | 1,102,451 |
| 3 | (“Dental Care for children” OR “Dental Health Services” OR “Oral health” OR "Dental Care" OR "Pediatric Dentistry") | 3,593 |
| 4 | ("Qualitative research" OR "Qualitative study" OR "Qualitative exploration" OR “Phenomenology” OR “Grounded theory” OR “ethnographic” OR “Action research” OR “Discourse analysis” OR “Focus groups” OR Interviews OR "Mixed-method approach") | 489,539 |
| 5 | S1 AND S2 AND S3 AND S4 | 45 |

**ProQuest: Dissertations and Theses Global**Date searched: October 14, 2022
Results retrieved: 482

| **Search** | **Search parameters** | **Results**  **Oct 14, 2022** |
| --- | --- | --- |
| 1 | (“Child” OR “children” OR “Preschool” OR “Infant” OR “Toddler” OR “minor”) AND (“Perception” OR “Acceptability” OR “Expectations” OR “Preferences” OR “Opinions” OR "Experiences" OR “Perspectives” OR "Parents views") AND (“Dental Care for children” OR “Dental Health Services” OR “Oral health” OR "Dental Care" OR "Pediatric Dentistry") AND ("Qualitative research" OR "Qualitative study" OR "Qualitative exploration" OR “Phenomenology” OR “Grounded theory” OR “ethnographic” OR “Action research” OR “Discourse analysis” OR “Focus groups” OR Interviews OR "Mixed-method approach") | 497 |

**Appendix S2:**List of excluded studies and reasons (n= 39).

| **Reason for exclusion: Phenomenon of interest (n= 26)** |
| --- |
| Badri P, Dahlan R, Amin M. Impact of Acculturation on Dental Attendance of Preschoolers Among Filipino Immigrants in Edmonton, Canada. Global Social Welfare. 2022;9(1):1–10.  *The complexity of the impact of social and psychological aspects of the acculturation process on the oral health of Filipino children in Edmonton.* |
| Barzangi J, Arnrup K, Unell L, Skovdahl K. Experiences and perceptions of infant dental enucleation among Somali immigrants in Sweden: a phenomenographic study. Acta Odontol Scand [Internet]. 2019;77(8):566–73.  *Experiences and perceptions of Infant Dental Enucleation (IDE) among a population of Somali origin living in Sweden.* |
| Burgette JM, Wu SX, Divaris K. “The pediatric dentist is different”: A qualitative study of young children's parents' experiences of oral health care in the Galapagos Islands. Int J Paediatr Dent. 2023 Jan;33(1):40-49.  *Caregivers' experiences and factors influencing their children's use of dental services on San Cristobal Island in the Galapagos Archipelago.* |
| Chi DL, Milgrom P, Gillette J. Engaging Stakeholders in Patient-Centered Outcomes Research Regarding School-Based Sealant Programs. J Dent Hyg [Internet]. 2018;92(1):16–22.  *Stakeholders and caregivers thoughts about a proposed patient-centered study to compare outcomes associated with silver diamine fluoride and resin sealants in school-aged children.* |
| Cortés DE, Réategui-Sharpe L, Spiro A, García RI, Cortes DE, Reategui-Sharpe L, et al. Factors affecting children’s oral health: perceptions among Latino parents. J Public Health Dent [Internet]. 2012;72(1):82–9.  *Parents' perceptions, experiences, attributions, and beliefs regarding their children's oral health, focusing on the role of social and psychological factors as potential sources of disparities in oral health.* |
| Custódio NB, Schardosim LR, Piovesan CP, Hochscheidt L, Goettems ML. Maternal perception of the impact of anterior caries and its treatment on children: A qualitative study. Int J Paediatr Dent. 2019 Sep;29(5):642-649.  *Parental perception about children's oral health-related quality of life (OHRQoL) following the rehabilitation of carious primary anterior teeth.* |
| Durey A, McAullay D, Gibson B, Slack-Smith LM. Oral health in young Australian aboriginal children: Qualitative research on parents’ perspectives. JDR Clin Trans Res. 2017 Jan;2(1):38-47.  *Perspectives and experiences of Aboriginal carers in terms of factors affecting their oral health and that of their children*. |
| Isong IA, Luff D, Perrin JM, Winickoff JP, Ng MW. Parental perspectives of early childhood caries. Clin Pediatr (Phila) [Internet]. 2012;51(1):77–85.  *Parents’ perceptions of caries influenced by their social and physical environment, and health influencing behaviours; how parents’ experiences and perceptions of caries influence ECC management and prevention at home, and parents’ knowledge and expectations regarding prevention and management of ECC.* |
| Kyoon-Achan G, Schroth R, DeMaré D, Sturym M, Sanguins J, Chartrand F, et al. Healthy Smile, Happy Child: partnering with Manitoba First Nations and Metis communities for better early childhood oral health. AlterNative [Internet]. 2021;17(2):265–74.  *First Nations and Metis (Indigenous peoples of mixed Indigenous-European, primarily French, ancestry) views on the challenges of keeping children caries-free.* |
| Kyoon-Achan G, Schroth RJ, DeMaré D, Sturym M, Edwards J, Lavoie JG, et al. Indigenous community members’ views on silver diamine fluoride to manage early childhood caries. J Public Health Dent. 2020;80(3):208–16.  *Understand Indigenous community members’ views on pediatric dental surgery to treat ECC under general anesthesia (GA) and receptivity to SDF as an alternative to restorative surgery. Treatments were not performed, only explained to the parents.* |
| Levin A, Sokal-Gutierrez K, Hargrave A, Funsch E, Hoeft KS. Maintaining traditions: A qualitative study of early childhood caries risk and protective factors in an indigenous community. Int J Environ Res Public Health. 2017 Aug;14(8).  *Perceived risk and protective factors, and overall experiences of early childhood nutrition and oral health in indigenous Ecuadorian families participating in a community-based oral health and nutrition intervention.* |
| Lima CMG de, Palha PF, Zanetti ML, Parada CMG de L, de Lima CMG, Palha PF, et al. Experiences of family members regarding the oral health care of children. Rev Lat Am Enfermagem [Internet]. 2011;19(1):171–8.  *Comprehend the experiences of family members regarding the oral health care of children, the meanings of oral health care, the search for the causes and prevention of oral diseases, and the reality of oral health services.* |
| Meyer BD, Lee JY, Lampiris LN, Mihas P, Vossers S, Divaris K. "They told me to take him somewhere else": Parents' experiences seeking emergency dental care for their children. Pediatr Dent. 2017 May 15;39(3):209-214.  *Caregivers’ experiences and perspectives related to seeking emergency dental treatment for their children.* |
| Muirhead V, Levine A, Nicolau B, Landry A, Bedos C. Life course experiences and lay diagnosis explain low-income parents’ child dental decisions: A qualitative study. Community Dent Oral Epidemiol. 2013 Feb;41(1):13–21.  *Better understand low-income parents' child dental care decisions through a life course approach that captured parents' experiences within the social context of poverty.* |
| Poirier BF, Hedges J, Smithers LG, Moskos M, Jamieson LM. Child-, Family-, and Community-Level Facilitators for Promoting Oral Health Practices among Indigenous Children. Int J Environ Res Public Health [Internet]. 2022;19(3).  *Facilitators for establishing oral health and nutrition behaviours for Indigenous children under the age of three.* |
| Raskin SE. Parents’ experiences with a school-based dental sealant project in central appalachia: A qualitative study. Health Behav Policy Rev [Internet]. 2020;7(3):215–22.  *Parents’ experiences with a rural school-based dental sealant project.* |
| Robles ACC, Grosseman S, Bosco VL, Couto Robles AC, Grosseman S, Bosco VL. Satisfaction with dental care: Qualitative study of the mothers of children treated at the Santa Catarina Federal University, Brazil. Cien Saude Colet [Internet]. 2008;13(1):43–9.  *Perceptions among mothers of the treatment provided through pediatric dentistry clinic and their concept of the ‘ideal’ dentist.* |
| Robles ACC, Grosseman S, Bosco VL. Practices and meanings of oral health: a qualitative study with mothers of children assisted at the Federal University of Santa Catarina. Cien Saude Colet. 2010;15:3271–81.  *Oral health practices and meanings of mothers whose children were attended in the dental paediatric clinics.* |
| Roguski M, McBride-Henry K. Insights into the oral health crisis amongst pre-schoolers in Aotearoa/New Zealand: A discourse analysis of parent/caregiver experiences. BMC Oral Health [Internet]. 2020;20(1).  *Parents/caregivers narratives to understand the barriers to engaging in effective protective behaviours.* |
| Smith PA, Freeman R. Living in a sweetie culture: Scottish parents’ difficulties in maintaining their children’s oral health. Health Educ J [Internet]. 2009;68(4):255–65.  *Experiences of living with a child with rampant tooth decay.* |
| Smith PA, Freeman R. Remembering and repeating childhood dental treatment experiences: Parents, their children, and barriers to dental care. Int J Paediatr Dent. 2010 Jan;20(1):50-8.  *Impact of childhood dental treatment experiences of a group of parents on the dental treatment they sought for their children who had dental decay and toothache.* |
| Vaughn HS, Robinson PG. The oral health-related experiences, attitudes and behaviours of the carers of Aboriginal children of Groote Eylandt. Int Dent J [Internet]. 2003;53(3):132–40.  *Exploring attitudes toward dental health, perceptions of barriers to receiving dental care, understanding of dietary factors, and current oral health status.* |
| Vermaire JH, Hoogstraten J, van Loveren C, Poorterman JHG, van Exel NJA. Attitudes towards oral health among parents of 6-year-old children at risk of developing caries. Community Dent Oral Epidemiol. 2010;38(6):507–20.  *Parents' prevailing attitudes towards the oral health of their children.* |
| Weinstein P, Troyer R, Jacobi D, Moccasin M. Dental experiences and parenting practices of Native American mothers and caretakers: What we can learn for the prevention of Baby Bottle Tooth Decay. ASDC J Dent Child. 1999 Mar-Apr;66(2):120-6, 85.  *Identify dental experiences, beliefs, and parenting practices of Native Americans to establish a preventive program.* |
| Wong D, Perez-Spiess S, Julliard K. Attitudes of chinese parents toward the oral health of their children with caries: A qualitative study. Pediatr Dent. 2005;27(6):505–13.  *Parents’ beliefs and perspectives regarding extensive caries (EC), oral hygiene and related habits, and dental treatment under general anaesthesia or conscious sedation, as well as reasons for refusing such treatment.* |
| Zolnikov TR, Garces K, Masood A, King R, Robbins M, McGuigan K. A mixed methods study reviewing consumer experiences for oral health treatment in Medicaid-eligible children in Florida. J Public Health Dent. 2022 Sep;82(4):365-371.  *Barriers to accessing and utilizing children's Medicaid oral health care and services, to evaluate care delivery and quality, and to assist in establishing a more consumer-driven approach.* |
| **Reason for exclusion: Study design (n= 4)** |
| da Silva BDM, Forte FDS. Access to dental treatment, mothers perception of oral health and intervention strategies in the city of Mogeiro, PB, Brazil. Pesqui Bras Odontopediatria Clin Integr [Internet]. 2009;9(3):313–9. |
| Newton T, Harris R. Summary of: What matters to patients when their care is delegated to dental therapists? Br Dent J. 2013 Mar;214(6):302-3. |
| Turton B, Durward C, Crombie F, Sokal-Gutierrez K, Soeurn S, Manton DJ. Evaluation of a community-based early childhood caries (ECC) intervention in Cambodia. Community Dent Oral Epidemiol. 2021 Jun;49(3):275–83. |
| Welbury R. Summary of: the development of a designated dental pathway for looked after children. Br Dent J. 2014 Feb;216(3):136-7. |
| **Reason for exclusion: Use of photos or videos (n= 3)** |
| Adams SH, Rowe CR, Gansky SA, Cheng NF, Barker JC, Hyde S. Caregiver acceptability and preferences for preventive dental treatments for young African-American children. J Public Health Dent. 2012;72(3):252–60. |
| Crystal YO, Kreider B, Raveis VH. Parental expressed concerns about silver diamine fluoride (SDF) treatment. J Clin Pediatr Dent. 2019;43(3):155-160. |
| Seifo N, Cassie H, Radford JR, Innes NPT. “I guess it looks worse to me, it doesn’t look like there’s been a problem solved but obviously there is”: a qualitative exploration of children’s and their parents’ views of silver diamine fluoride for the management of carious lesions in children. BMC Oral Health [Internet]. 2021;21(1):367. |
| **Reason for exclusion: Findings without illustration or not properly identified (n= 6)** |
| Akera P, Kennedy SE, Schutte AE, Richmond R, Hodgins M, Lingam R. Perceptions of oral health promotion in primary schools among health and education officials, community leaders, policy makers, teachers, and parents in Gulu district, northern Uganda: A qualitative study. PLoS One. 2023 Nov 2;18(11):e0293761. |
| Chang CP, Barker JC, Hoeft KS, Guerra C, Chung LH, Burke NJ. Importance of content and format of oral health instruction to low-income mexican immigrant parents: A qualitative study. Pediatr Dent. 2018 Jan 1;40(1):30-36. |
| Dyer TA, Owens J, Robinson PG. What matters to patients when their care is delegated to dental therapists? Br Dent J. 2013 Mar;214(6):e17. |
| Fleming PS, Colonio-Salazar F, Waylen A, Sherriff M, Burden D, O Neill C, et al. Prioritising NHS dental treatments: a mixed-methods study. Br Dent J. 2022 Jan. |
| Piggott S, Carter S, Forrest H, Atkinson D, Mackean T, Mcphee R et al. Parent perceptions of minimally invasive dental treatment of Australian Aboriginal pre-school children in rural and remote communities. Rural Remote Health. 2021 Nov;21(4):6862. |
| Quintero M del CV, Cerezo Correa M del P, Cifuentes Aguirre OL, Paz Delgado AL, Parra Ramírez G. Systematization of the healthy smiles program implemented in Manizales, Colombia. Rev Cubana Estomatol [Internet]. 2020;57(2):1–14. |

**Appendix S3:**Synthesised findings

| **Synthesised finding 1: Family-centred care components identified in the dental attendance – 44 findings (43U, 1C)** |
| --- |
| **Category 1: Trust in the professional (n=21 – 20U, 1C)** |
| **Most parents were accepting of the treatment primarily because it was recommended by the dentist to manage their children’s ECC and they were trusting of the dentist’s recommendation. (U)**^23^  “We never have seen before this kind of treatment. The doctor explained to us and so we were satisfied about his explanation. That’s why we’ll go with that one.” ^(p. 4)^ |
| **Parents felt comfortable with the information being delivered by health visitors and thought they were the right person to deliver the intervention. (U)**^15^  “Obviously, you get a bond with them, don’t you, cause they’ve, like, you know, measured your baby and they’ve measured their everything, their head, this and that, and they come round from when they’ve been little for, like, now. So, you’re more comfortable, aren’t you, with them.” ^(p. 5)^ |
| **Having a “personalised” visit is essential for parents and strengthens the notion of having a good rapport and establishing “bonds”. (U)**^15^  “It’s just cause if you have previous concerns you’ve already spoken with the health visitor about and if you see her again she’s like “aw well has that improved?” cause she’s already seen you before. And she’s said you know she’ll recap on the last meeting and it feels more personalised and it’s a lot better cause then they know that child. And if there were concerns or things that stood out to them in one meeting they can sort of look at it in the following meeting and have a look whereas if it was someone different they would just look at it as if it was a new child and previous notes.” ^(p. 6)^ |
| **Parents felt that the familiarization the dental setting was effective in reducing their child’s dental fear and anxiety. (U)**^16^  “She knows that no one is going to hurt her [during the oral examination] and she is not going to have any pain, and then she is comfortable. And it is good that she is comfortable because if we come back for the operation [and she is not comfortable] then she won’t sit on the chair.” ^(p. 410)^ |
| **Trusting the DP to make the right decision was a significant factor in parents’ acceptability. (U)**^19^  “… the fact I trust [DP name] …. She’s very clear, she explains things very … and really takes the time both with [child’s name] and me …. And that helps I think to make a decision.” ^(p.7)^ |
| **Continuity of care with the same DP and regular visits was important in allowing the DP to gain the trust of the child and parent. (U)**^16^  “She’s very patient with [child’s name], particularly with the children in helping her to understand. When she first came she was very reluctant to even open her mouth. … … … ..We’ve been coming here forever.” ^(p. 10)^ |
| **Parents who trusted the DP were confident that their child was being well cared for; Thus, the treatment alliance may be built between the DP and the child through the personal beliefs and experiences of the parent. (U)**^19^  “She knows what she’s doing and she takes care of my teeth … I mean my teeth were a disgrace when I went to her. And she’s fixed them all up and I’ll just go and say, “What do you think?” And kind of whatever she says, I’ll go with it. If she was to say, “Oh yeah it needs to come out, it’s going to cause a problem,” then we would do it.” ^(p. 10)^ |
| **Parents found the conventional approach acceptable, and this was strongly associated with trust in the DP providing treatment. This trust enabled a treatment alliance between the child, parent and DP and facilitated the acceptance of the treatment by the child. (U)**^19^  “It’s a bit painful when they do that, injection. That’s why he was a little bit scared. But he was fine. I was very happy with everything”. ^(p.6)^ |
| **Engaging with parents personally, allowing them the opportunity to support their child while undergoing treatment was also appreciated. (U)**^19^  “[DP name] explains everything really well. And like she’s very big on pull a chair up, hold her hand, have a look at what we’re doing”. ^(p.10)^ |
| **Acceptability of procedures was linked to trust and the building of a treatment alliance between the child, the parent and the DP providing care. The patient management skills of the DP were able to facilitate a trusting relationship which had a positive impact on compliance and reducing dental anxiety in both child and parent. (U)**^19^  “When before appointments, she was crying and everything, but when the dentist was suggesting us to do it in front of her and she was listening to her, and then when we’re coming and she was saying, okay, you’re allowed to do it just because of the dentist”. ^(p.7)^ |
| **Despite anticipating their child’s non-cooperation, parents found that the dental team was usually able to facilitate the child’s acceptance of the procedure. Trust in the DP providing care was a significant factor in managing dental anxiety. (U)**^19^  “We’re very lucky because she really, really likes ‘the dentist’, don’t you? …. She’s made you feel really, really comfortable. Sometimes even if when you’re feeling a bit nervous, she’ll still get in the chair and at least let her look and things”. ^(p.9)^ |
| **Parents believed their children’s oral health-related knowledge improved because it was imparted by a professional, i.e., a dentist. (U)**^17^  “Young children may need someone else, not their parents or family members, to tell them what they need to do or to improve because they would like to listen to teachers and dentists.” ^(p.3)^ |
| **Children – at least as much as parents – need to take responsibility for their own oral health. (U)**^16^  “She [the dental professional] didn’t just talk to me, she made sure that they’re involved [the child]. And they [the child] have to take care of their teeth as well as mum and dad.” (p.415) |
| **Parents highlighted how the child could be actively involved when the advice is communicated. (U)^13^**  “If they notice a build-up of plaque anywhere then they will say you know you should be focussed on these areas. I would say my older boy has had x-rays and things recently and the dentist really had a good talk to us with that and like look at the x-ray and got him really quite involved with it which was really nice for him.” ^(p.4)^ |
| **All parents learning about SDF as a treatment option for caries from the dentist in the study. (U)**^23^  “I asked the doctor and he explained. He said that it is safe so we’re not worried about it (…) A lot of time at the dentist and he said, this one will help me to stop cavities and the cavities was causing too much problems. It helps a lot.” ^(p. 4)^ |
| **One parent specifically commented that the dentist first introduced her to SDF when she brought her child in for a first visit, which happened to be a dental emergency.** **(U)**^23^  “He [child] had tooth pain in the back there. And the doctor actually had to remove his tooth that day. We went in . . . for an emergency dental appointment, he pulled his tooth out. He [doctor] talked to me about the silver treatment because my kids have never been to a dentist before.” ^(p. 4)^ |
| **Not having prior knowledge of the product, some parents were happy to have received additional information and articles to read about SDF to make an informed decision so that they were not just taking the dentist’s word of the effectiveness of the treatment. (U)**^23^  “He [dentist] said it is safe. So I trust him. He gave us a few papers to read about it. I read all of those and I think it’s good.” ^(p. 4)^ |
| **For the parents/caregivers who had their child managed under ART/HT, they were pleasantly surprised, not only that treatment was able to be done but the extent of the treatment, which sometimes included extractions. (U)**^12^  “The school dentist wouldn’t touch her because she is terrified of the dentist . . . a private dentist and they wouldn’t touch her, so thank God for (clinician’s name) . . . and (child’s name) settled within the first two visits. I could not believe she had done all that work and (child’s name) just laid there and let her do it. It was amazing.” ^(p.8)^ |
| **General dentists were not used to treating child patients, and mostly they opted for extractions of primary teeth when reported with pain. They did not advocate preventive procedures also. (C)**^27^  “My daughter's back tooth was removed long back due to tooth decay… The doctor did not give any clip (space maintainer) for that area, and he did not mention anything related to that. He insisted that permanent tooth inside will erupt eventually. It has been a year, but no sign of permanent tooth is seen”. ^(p.219)^ |
| **The opportunity to be referred to a child-friendly dentist was a highly valued element of the intervention, particularly to those participants who had specific needs. (U)**^22^  “I was expecting her to go away and say “oh they said no”. But to be referred to the dentists and that was really good and she was just genuinely really friendly”. ^(p. 141)^ |
| **Lack of interdisciplinary practices among paediatricians, physicians, general dentists, and paediatric dentists also influenced the child's dental care. (U)**^27^  “We used to meet our daughter's physician during every vaccination schedule, but he never asked us to have dental check-up; neither we enquired about her tooth problems”.  ^(p. 219)^ |
| **Category 2: Professional communication skills (n=8)** |
| **Parents did not want to be ‘lectured’ about their child’s oral health. (U)**^16^  “I thought I was going to get drilled with, you know, you shouldn’t be doing this, you shouldn’t be doing that, but they have been pretty good with everything, you know (…) they were offering suggestions, and they were coming from experience some of them with the kids as well, rather than giving you unrealistic goals.” ^(p. 415)^ |
| **Participants’ appreciation of a non-confrontational, patient-centred style of parent counselling seemed to be influenced by their feelings of guilt and/or embarrassment about the condition of their child’s carious teeth. (U)**^16^  “When I came in I was like ‘oh here we go, everyone’s going to think I’m a bad mother’. That’s what I was thinking. When I look at him now I think shit, why did I give him that bottle? I feel guilty.” ^(p. 415)^ |
| **Some parents reported how the preventive intervention enabled a two-way, friendly conversation between parent and dental team members. (U)**^14^  “What was helpful for me was that I went in thinking, ‘I need to come out with a list of things that they’re not allowed to have, and I was ready for a bit of a bashing [laughs]. Whereas actually, that wasn’t really what I came out with. It was more like treats are okay, […] but just be careful and think about when you’re giving them to them and make it work for your family rather than restrict [them].” ^(p. 4)^ |
| **Parents reported that their child’s cooperation with treatment was positively affected by the skills of the DP: their ability to make children feel comfortable and less anxious was important for parents. (U)**^19^  “She likes [DP name] so much … .so she wants to be there all the time she said as well … And she’s quiet as well when she gets this treatment done”. ^(p.9)^ |
| **Empathy shown by the DP when caring for their child, was important for developing the treatment alliance and having a positive impact on acceptability. (U)**^19^  “If he wasn’t happy, he would cry. I would know if he’s unhappy or he’s upset. So he’s never, yeah. … She’s good with him. And he kind of like listens so... It’s her who speaks to him gently and he is listening and yeah”. ^(p.10)^ |
| **Empathy was described as an attribute of a ‘good’ DP. Conversely, a DP that was ‘not good’ lacked empathy when caring for their patient. (U)**^19^  “Shows concern. it helps if you’ve got a bit of time; ask them what they’re doing at school”. ^(p.10)^ |
| **Parents expressed appreciation of the concept of child-centred/family-centred care when it was experienced. (U)**^12^  “Yeah, and I think the way (clinician’s name) did it, she said, “Well, we’ll work up to scary things like pulling the most painful teeth out”. So, all that was done at the end when she was quite confident to go in. So, she started off with the smaller things and then worked up to fillings and things like that… Yeah, so dentist visits are now viewed as a positive experience… Everyone involved was very approachable and non-judgmental…, ever since she was young, every time we went to see the dentist, they sort of made us feel like we were doing something wrong with her and that’s why her teeth were like that… it didn’t feel very rushed; … “It’s a trust, yeah, to build that rapport.” ^(p.5)^ |
| **Some mothers emphasized the importance of face-to-face motivational interviews sessions for motivation. (U)**^21^  “The interview is the best”. ^(p.6)^ |
| **Category 3: Oral health guidance and supporting materials (n=15)** |
| **Participants appreciated the face-to-face demonstrations that they received from the dental professional. (U)**^16^  “It helped me a lot. I know how to brush correctly for them. It’s helped me for the first child and the second child.” (p.412) |
| **Parents highlight the importance of visual demonstrations to help identify problematic toothbrushing techniques, and the positioning of these resources are important to encourage oral health conversations. Parents stated how they recognised their toothbrushing techniques were wrong after they had seen the demonstration. (U)**^14^  “Once you start getting children, or even yourself, for it to be mandatory for someone to show you how to brush your teeth.” ^(p. 7)^ |
| **Parents found the preventive strategy to be beneficial to them as a parent in terms of encouraging ways to improve tooth brushing. (U)**^19^  “Spent quite a lot of time on helping us to brush properly (…) she’s very good in terms of giving us advice in terms of how to brush and obviously looking at the pink and knowing where we’re missing, that helps as well.” ^(p.8)^ |
| **The leaflets supported parents to have oral health conversations with wider friends and family, especially where they were previously hesitant to do so. (U)**^14^  “[the leaflet] says you should do it twice a day. Cause that argument of well yeah I only want to do it once, it’s like, ‘well actually, the guidelines suggest…’. So it’s nice to have a bit of, for me, yeah a bit a’ back up… I can reinforce the message that I’m already saying. Cause people just think that I just, I’m saying it for no reason. So I feel like if its written down and published, if somebody paid to get it printed they might listen a bit more.” ^(p. 7)^ |
| **The leaflet also provided parents with the opportunities to pass on information to the wider family. (U)**^15^  “But they concentrated a lot more on the toothbrushing, which was really good. So it was good for me cause I know how important it is but you don’t know how to explain to other people. Sometime like my mother-in-law or someone [unclear words 0:05:03]. So, it was really good the way they explained it so I could tell my mother-in-law, ‘look’. And I gave her that little booklet as well and I said, ‘look this will help’. So, it was really good.” ^(p. 9)^ |
| **“The “Strong Teeth” resources received positive feedback from participants who discussed how they facilitated oral health conversations, not only between parents and the wider dental team, but also between friends and family.” (U)**^14^  “The leaflet for the family just reinforces what I tell her dad and my parents in official text. I think that did encourage us.” ^(p. 6)^ |
| **One parent highlighted the likelihood of leaflets being discarded if they were to be provided at the end of the visit. (U)**^14^  “Even if you were given the leaflets, it’s not gonna encourage you to read them. It’s gonna be something that you stuff in your bag while you’re trying to pick your kids up and that you probably don’t end up looking at. Whereas when she’s actually sat down, and she’s going through it with you, I think you’re more inclined to ask questions and understand more.” ^(p. 7)^ |
| **Many dental team members gave more than two leaflets within their appointment. This meant that a wide range of topics were covered and could have led to ‘information overload’. This made it difficult for parents to remember the context of the resources. (U)**^14^  “I think there were 3 [leaflets] … One of ’em was definitely about foods and stuff; I can’t remember now it’s been a while.” ^(p. 8)^ |
| **Participants suggested that receiving information during prenatal care can be more effective than during postnatal care. (U)**^SCHROT^  “When you’re in the hospital they give you quite a few pamphlets on different things. Maybe that’d be a way to get the message out too. But pediatricians regularly see babies from six months, so maybe they should be the ones that are promoting that FFV and giving pamphlets out at your first appointment around the six months [visit].” ^(p.5)^ |
| **Beyond social and family contacts, the guidelines received in the maternity ward were valued and incorporated into the care of their babies. (U)**^20^  “Just in the maternity hospital, we learned how to wipe a baby's mouth. In the maternity, it is good! There you learn everything”. ^(p. 672)^ |
| **The mothers had positive perception of the components of intervention, expressing that they were satisfied with them, and that the interventions motivated them to brush their children’s teeth. (U)**^21^  “When you send me something occasionally, and I see it, it makes a difference and encourages me to brush their teeth”^(p.6)^ |
| **Mothers described that the components of intervention acted as reminders to brush their children teeth. (U)**^21^  “It is something that makes me remember and reminds me when I forget”. ^(p.6)^ |
| **The mothers commented on how the story telling videos were relatable and similar to real life experiences. (U)**^21^  “My son Mohamed, for example, is exactly like the boy in the video”. ^(p.7)^ |
| **The mothers felt that the stories depicted in the videos were authentic and genuine, and some shared personal experiences. One mother recounted an experience she had with a child who suffered from stuttering. She felt that witnessing such tangible stories would convince many mothers to take immediate action and take care of their children’s teeth. (U)**^21^  “By the way, the story of the stuttering that I saw in this video… I had a personal experience with a child who was the same way, and his front teeth were all decayed like that, and the way he spoke, even though he was somewhat older…… It was very clear to us that he was embarrassed. This is tangible and is something that would make any mother take care of her child’s teeth immediately”. ^(p.7)^ |
| **For parents, there appeared to be little recollection of what was discussed within the dental visit. (U)^13^**  “At the time they told me what fluoride content to look out for but I can’t remember now.” ^(p.3)^ |

| **Synthesised finding 2: Aspects of dental interventions and settings that facilitated their acceptability – 31 findings (31U)** |
| --- |
| **Category 1: Positive characteristics of minimally invasive dentistry (n=18)** |
| **Their preferences also appeared to be affected by personal past experiences. (U)**^19^  “I prefer the preventative, because I’ve had fillings and I didn’t like getting them at all. And that kind of puts the fear in. So, if we can stop getting fillings, then, we can stop the fear.” ^(p. 7)^ |
| **The service provided treatment for dental caries and prevented the further development of dental caries, which in turn improved oral health of their children. (U)**^17^  “At least he could receive some treatment. Or maybe the treatment could prevent further decayed teeth? I am not sure. But at least he received something and it should be beneficial to his oral health.” ^(p. 3)^ |
| **Some parents were accepting of SDF treatment because it is a painless procedure. (U)**^23^  “I think that this is good for the kids because it is a painless treatment.” ^(p. 4)^ |
| **Parents reported acceptance because the treatment (SDF) was minimally invasive, and avoided the need for surgical intervention with the dental drill and restorations. (U)**^23^  “I don’t want to go through like all those extensive procedures.” ^(p. 4)^ |
| **The majority of parents preferred SDF treatment to more invasive techniques. (U)**^23^  “In my opinion, this [SDF] is better than the filling because it’s not a painful process.” ^(p. 5)^ |
| **Some parents were accepting of SDF treatment because it decreases sensitivity and halts the progression of the child’s caries lesions. (U)**^23^  “It reduces the cavity (…) When we use SDF on the teeth, after a few minutes, pain stopped.” ^(p. 4)^ |
| **Some parents accepted SDF therapy even though it caused black staining on the treated lesion because oral health is valued more than aesthetics. (U)**^17^  “I knew SDF would cause black staining and I was worried but I still joined this service because I did not want my daughter’s tooth decay to get more and more severe. If the decay progresses and reaches the root, it will be too late.” ^(p. 6)^ |
| **Parents whose children received the SDF treatment expressed that non-GA pathway options should be promoted more often when managing caries. (U)**^24^  “I am glad that we did this [SDF]… I feel better knowing that she didn’t have to go under… The only thing is that her teeth [are] going to be black now, not the whole tooth but you know just that little part. But I’m glad that it’s going to stop the cavities.” ^(p.6)^ |
| **Almost all parents in the study said that they would recommend SDF to other parents as a way to nonrestoratively manage ECC. (U)**^23^  “I recommend it to the other people. It is a good treatment for the kids because it is painless. Everything is good, that’s why.” ^(p. 6)^ |
| **Some parents said that they had already recommended the treatment (SDF) to their family members and others. (U)**^23^  “I already informed other parents who have the same problem like my child because this treatment is helpful. When you see a child, a small child having his teeth decayed and broken and going to be worse, you’re worried. But when I see this medication and [how] it comes to normal, that is where I already told some parents.” ^(p.5)^ |
| **Parents shared that additional information should be provided to parents/grandparents and other caregivers so that they can be made aware of all the treatment options available for their children. (U)**^24^  “They never heard of anything like this. Most of the time so far, almost everybody I’ve told [about the SDF treatment] they were really surprised . . . and they liked that idea instead of having their kids to be put under or you know, having to watch their kids go through all of this pain and yeah.” ^(p.7)^ |
| **Parents found the biological strategy acceptable and reported that they would agree to undergo the procedure again if needed. Parents also expressed the value they placed on avoiding any drilling and injections. (U)**^19^  “I’m very glad she got this one …. if someone came near her mouth with the drill, she wouldn’t be happy at all”. ^(p.6)^ |
| **Some parents reported that their child was not concerned about local anaesthetic injections before treatment. (U)**^25^  “He is not anxious…he has had good experiences in the past.” ^(p.15)^ |
| **Parents were relieved when teeth could be treated more conservatively, and the extractions were able to be avoided. (U)**^12^  “I was told initially, they would just pull out as a precaution are now still in place and looking very healthy (…) and he has none removed at the moment. She did say we don’t know about the molars whether they will have to be eventually but I think three other teeth were saved.” ^(p.8)^ |
| **Parents/caregivers who have had previous experience with a general anaesthesia and were managed through the ART/HT were very satisfied with the care received and even expressed a willingness to pay for the care. (U)**^12^  “. . . either way we are going to have to pay. . . . so to be able to pay for treatment that he has in the chair and he still comfortable and confident to go to the dentist I think it’s all you need.. . . I just feel so much better knowing that he has had the treatment that he needs without having to be completely knocked out. . . .” ^(p.8)^ |
| **Some parents did not have any aesthetic concerns regarding PMCs. (U)**^19^  “It seemed sensible …. don’t really care what the look of it is”. ^(p.6)^ |
| **Opinions of the Hall Technique were very positive. (U)**^25^  “You don’t have to go through all the injections… they’re good to go within minutes.” ^(p.15)^ |
| **There were also some concerns about the procedure, crown size, the metals used in the crown and its longevity. (U)**^25^  “It will stay there for the life of the tooth… is that right?”. ^(p.15)^ |
| **Category 2: Aspects related to dental services (n=9)** |
| **Some parents indicated that finances were limited and anything to offset the cost was appreciated. A few said they went earlier than they otherwise would have because it was free. (U)** ^SCHROT^  “I wouldn’t have gone as early if wasn’t free. So it encouraged me to go and see why they want them to go at one, or one and a half.” ^(p.4)^ |
| **Although not all dental needs were being addressed by the FFV program, the overall impression in the focus groups was positive. (U)** ^SCHROT^  “I think too, that by having this program there would be a lot more people that would take advantage of it, and it’d give the dentists a lot better idea of what stages people’s teeth are at, or what problems they need, things that they could make better and that by seeing more children through this type of program, maybe more people would come that wouldn’t have come before.” ^(p. 5)^ |
| **Cost of dental care was also a strong theme from parents/caregivers after the specialist consultation in an oral health centre. (U)**^12^  “I would have delayed completing the treatment . . . because money was quite tight...” ^(p.7)^ |
| **Parents/caregivers were able to compare the ease/difficulty in physically getting to the location for their child to be treated. The principal issues identified were the distances involved in getting to the oral health centre, which is in the city. (U)**^12^  “. . . . . . I didn’t have to travel one and half hour each way to oral health centre. . . . . .” ^(p.7)^ |
| **Some mothers tried to find a solution to attend interventions. (U)**^21^  “I have two days off per month and can use these to come here to gain experience”. ^(p.7)^ |
| **Some stay-at-home moms felt they could allocate some free time to receiving the interventions.** (U)^21^  “I am a housewife and I have a lot of free time, so I don’t feel like it troubles me”. ^(p.7)^ |
| **The mothers explained that the online interventions were easily accessible, and no effort would be exerted to participate in the interventions using them. (U)**^21^  “I hold my mobile phone all day, so there is no effort”. ^(p.7)^ |
| **The mothers highlighted that almost all people nowadays have internet connection at home, and there was no addi­tional cost associated with the online interventions. (U)**^21^  “There is no [additional] cost. We hold the mobile phone in our hands all day long. Instead of playing games, it is better to see something useful for a change”. ^(p.7)^ |
| **Participants also felt the importance and usefulness of sharing dental health information on social media plat­forms, since technology is the main method of commu­nication between people nowadays, regardless of their social and educational level. (U)**^21^  “I wish you would upload the videos on the internet so that mothers could learn how to deal with their daughters and brush their teeth. Encourage the mothers; there are moth­ers who do not know [how to take care of their children’s teeth]”. ^(p.7)^ |
| **Category 3: Dental care in the school environment (n=4)** |
| **Several parents thought that their children behaved better, made less fuss or were less nervous about seeing a dental clinician in a school environment. (U)**^18^  “Interviewer: So he’s a bit better in school? Parent: Yeah, as the teachers are there. Interviewer: And you’re not? Parent: That’s right he’s a good boy in school see [laughs] it’s an awful thing to say but it’s true”. ^(p. 95)^ |
| **Parents trusted the school and several said that they participated partly because of this. (U)**^18^  “It was with the school I was quite happy because it was recognised through the school.” ^(p. 99)^ |
| **Dental care in kindergarten could alleviate the child’s dental fear and anxiety and allowed them to adapt to dental examinations. (U)**^17^  “Young children will be scared when they go to a dental clinic but they may have a good experience if the dental check-up is at kindergarten. Everyone in the kindergarten opens his/her mouth and the dentist uses a cotton bud to check everyone’s teeth. It looks funny and so the children feel like playing. It will be good training for them. … and if the parents are not around, they (the children) need to grow up and rely on themselves. If the parents are present, they may cry and not want to see the dentist.” ^(p. 4)^ |
| **Parents reported that awareness about the first dental visit has not reached them so far and added a few points regarding the changes to be done in the society to spread this awareness. (U)**^27^  “Nowadays, kids are learning a lot in schools. The importance of primary teeth and first dental visit can be added in school syllabus. Then both parents and children will get to know more about this”.  ^(p. 219)^ |

| **Synthesised finding 3: Influence of oral health conversations and instructions on parents’ behaviour – 34 findings (34U)** |
| --- |
| **Category 1: Impact of the received information (n=14)** |
| **Parent discusses how they would have liked to have the knowledge of how to perform toothbrushing correctly before their child’s first tooth had erupted. (U)**^15^  “…when she come out, he had his first tooth. So, he were a bit earlier with his teething, but, see he got his first tooth before six months, and I think the four, five, six-month mark would probably be better [for a visit]. If I had earlier, I wouldn’t have done what I were doing in the first place.” ^(p. 8)^ |
| **Mothers, regardless of experience, stated how they were previously unsure of when to begin toothbrushing and for how long they should brush their child’s teeth. (U)**^15^  “…I suppose just the information about if there’s anything, in particular, you need to look out in terms of toothbrushes and toothpaste and things like that. Confirmation that you should just start when you should start brushing them.” ^(p. 6)^ |
| **Although some parents perceived additional information as unnecessary and non-essential, they stated how the intervention had provided reassurance** **that they were undertaking appropriate oral health behaviours. (U)**^15^  “They give you reassurance that he’s following the right stages cause maybe as a first-time parent, or a second time or even a third time just check... is he doing okay, is this normal so kind of ask the first point of call is “is this normal?” ^(p. 6)^ |
| **One parent wanted to ensure she had done enough to clean her child’s teeth without causing too much distress. (U)**^15^  “Maybe a bit more, well what’s the minimum I need to do? Literally get some fluoride on the teeth.” ^(p. 8)^ |
| **Parents shared what they learned through their experiences and were committed to not making the same mistakes again. (U)**^24^  “I’m hoping some will relate to it and not feel so I guess bad, or you know? No parent wants to see their kids or hear that they need to go under and go in for surgery. So, I am really hoping that it does help them.” ^(p. 7)^ |
| **The parents believed that this service** **helped them to know more about their children’s oral health status. (U)**^17^  “I noticed she had two decayed front teeth but I did not know much about it. Would it get severe? Would it hurt? Or whatever, I did not know. So, I would like to join this service and let the dentist take a look. Then the dentist can let us know how the tooth decay will progress.” ^(p. 3)^ |
| **The parents considered the parental education seminar to be useful. It provided essential knowledge to them about how to identify oral health problems in their children and what they should do to protect their children’s teeth. (U)**^17^  “I did not realise my child had tooth decay. I thought it was just a little bit dirty on the tooth surface. If the dentists did not tell me, I would never know. But after joining this service, I know this is tooth decay.” ^(p. 5)^ |
| **Most parents participating to generally benefit their children’s teeth and to feel that they had done as much as possible to promote their child’s dental health. (U)**^18^  “I’m thinking myself there’s every chance now that I’ve given her the best chance to look after her teeth then and she may not have decay.” ^(p. 98)^ |
| **Some parents wanted to monitor the progress of their child’s tooth decay and therefore valued the feedback that they received from the child’s dental assessment. (U)**^16^  “Here they check her teeth properly and I want to know if they have gotten worse or stayed the same.” ^(p. 410)^ |
| **The parents noticed that the outreach dental service allowed their children to understand their own oral health status. (U)**^17^  “The annual dental examination is like an annual review. My child put effort into brushing better and maintaining better oral health because he wanted to get better results in the next examination.” ^(p. 3)^ |
| **Some parents said they learnt from this service that regular dental visits are necessary even for young children. (U)**^17^  “This service lets me and my child know that if we have tooth decay, we have to go to see a dentist and seek treatment. Even if we do not have tooth decay, we still need to see a dentist for regular check-ups.” ^(p. 5)^ |
| **The interventions also provided confirmation supporting them to take care of their children’s health. (U)**^21^  “I feel like I know this information, but I feel you gave me a push to take care of their teeth and honestly, I am happy”. ^(p.6)^ |
| **The mothers stated that understanding the potential harm resulting from poor oral health had a stronger motivational effect compared to simply hearing a story. (U)**^21^  “The harm, if it is more, is more motivating than having one tell a story, because the harm is what makes mothers more motivated”. ^(p.6)^ |
| **The mothers also highlighted the importance of focusing on the negative consequences of caries on various aspects of their child’s life. (U)**^21^  “The impact on eating, school, and concentration because of cavities, …. [should be highlighted] … because school is very important”. ^(p.7)^ |
| **Category 2: Improvements in health-related behaviours (n=20)** |
| **Among parents who reported a reduction in their child’s dental pain and a subsequent improvement in the child’s quality of life, a number felt that the improvement had a positive impact on their own emotional well-being. (U)**^16^  “She sleeps more soundly and she doesn’t complain. It makes us happier. There’s less stress.” ^(p. 414)^ |
| **Parents reported a reduction in the frequency and intensity of the child’s dental pain by increasing the regularity and quality of tooth-brushing. (U)**^16^  “During the waiting time I brushed for her and her disease it’s gone and her dental pain too. I brushed for her, and in about one week, all the pain had gone, and then she was sleeping better and eating better. Before, she didn’t want to eat.” ^(p. 413)^ |
| **The parents agreed that their children’s toothbrushing behaviour improved after the service. (U)**^17^  “My boy did not brush very well before. He did not know where to put the toothbrush and how to brush, and he did not like toothbrushing at all. But after joining this service, he brushes more carefully and for longer. He told me that the dentist asked him to do so.” ^(p.3-4)^ |
| **Parents mentioned that their oral health care routines with their children had either changed or improved since the children had undergone the dental procedures. (U)**^24^  “Now they are getting their adult teeth and I’m like these are what you are going to have for the rest of your life, so you need to take care of them. (…) They improved some with having their teeth brushed. (…) I don’t want them to go through pain and dental problems.” ^(p. 6)^ |
| **Some participants felt that children would brush their teeth with the desired frequency if a regular ‘routine’ time for brushing was established. Some reported that** **linking tooth-brushing with established household activities (such as bathing and dinner) was a useful way of establishing a routine. (U)**^16^  “It just seems to be a natural routine now that after dinner they go in and brush their teeth”. ^(p. 413)^ |
| **Parents reported an overall improvement in their child’s oral health with changes in their oral health behaviours, including improved tooth brushing and reduced sugar intake. (U)**^19^  “And about fizzy drinks, they’re all quite conscious about they’ll go “Oh, [DP name] says but only as a treat.” [DP name] does it in a nicest possible way, but in a way that they remember so. And it’s good for them especially as they’re getting older.” ^(p. 9)^ |
| **Parents preferred avoiding fillings and found other aspects of the prevention arm a positive experience. (U)**^19^  “I’d say a lot of positive things has come out of it. There’s nothing negative, definitely something positive. And it makes the children aware (…) What they’re eating and what they’re doing...I think it’s been really helpful.” ^(p.7)^ |
| **The intervention was welcomed by most of the participants, and many commented about and were pleased and proud of the changes in their behaviours which it had stimulated.** **(U)**^22^  “It’s gone from having breakfast to now sitting down having breakfast, brush her teeth, carry on her day, bedtime milk, brush teeth and then bed so obviously the routine changed”. ^(p. 140-1)^ |
| **Parents/caregivers reported a broad range of impacts, which ranged from pain and functional limitations to psycho-social distress, experienced before treatment, not only by their child but also by themselves because of the ECC. (U)**^12^  “. . . like my daughter before is very bad pain have, and all the time he cry, miss the school, all the time not eating good, every time he’s eating – all night I wake up just give him like . . . and all the time I can’t sleep, myself, like I’m tired because when I see my daughter all the time cry . . . He’s weight goes to 12 kilos”. ^(p.4)^ |
| **Parents/caregivers reported improvements in children's health after treatment and in their well-being. (U)**^12^  “After treatment now I’m very happy like normal.. . . Yes it’s now good, now 22 kilos”.^(p.4)^ |
| **Children and parents across all strategies reported a positive impact in terms of less pain and improved oral health. (U)**^19^  “Yes, it seems fine. And we have no complaints, no problems, no toothaches with it …. she doesn’t complain when she’s eating or anything anymore”. ^(p.9)^ |
| **Having older children with an established routine with regards to oral health was a facilitator to parents taking on board messages from the HABIT intervention. (U)**^15^  “I think with having [name of older child], I don’t know, I think for me, I just, I dunno, you know to brush their teeth. So as soon as both of them got teeth, it was just an obvious thing.” ^(p. 9)^ |
| **The desire to copy older siblings could make young children resistant to receiving direct parental instruction, but they overcame this problem by providing direct, hands-on supervision either before or after allowing the child to brush unassisted. (U)**^16^  “She wants to do it by herself but she can’t do it properly because she’s little. She starts, and then I take the brush and then I do it.” ^(p. 413)^ |
| **Young children often wanted to copy the behaviour of their siblings, especially older ones, which could lead to the enthusiastic uptake of tooth-brushing. (U)**^16^  “They are encouraged to brush their teeth because they see their older brother doing it, so it’s good”. ^(p. 412)^ |
| **Participants felt that the taste of toothpaste could influence brushing behaviour and that certain oral hygiene products appealed to children and could play a role in encouraging brushing – such as battery-operated toothbrushes and dental products that carry the name of popular child television characters. (U)**^16^  “She says ‘No daddy I don’t want that one, I want the small one with the Wiggles on it’”. ^(p. 413)^ |
| **Participants highlighted how the Oral-B electric toothbrushes made toothbrushing “fun” for the child and appeared to motivate them to use their Oral-B electric toothbrush (perceived effectiveness).** **(U)**^14^  “There was some that were just like so excited to come like oh I’m getting the electric toothbrush OMG [“Oh My God”].” ^(p. 6)^ |
| **The narrative highlights that parents are willing to buy appealing products, such as electric brushes if they were motivated to do so and could see the benefits of their purchase. (U)^13^**  “Yeah, yeah I mean we got the Star Wars flashing Lightsaber one that kind of gives you a time limit of 2 minutes. It flashes for 2 minutes and makes noises for 2 minutes so yes that worked until it broke but it’s very expensive so we didn’t get it again but it did make him respond.” ^(p.6)^ |
| **‘Brushing rewards sheet’ was effective in encouraging children to brush. (U)**^16^  “She was going in and cleaning her teeth twice a day and going in and filling it in and colouring it in and receiving a reward at the end of the week . . . it makes your job a little easier to try to keep her teeth clean.” ^(p. 412)^ |
| **Parents said that children found it novel and exciting to visit the MDC (known colloquially as ‘the van’) and that they liked various aspects of treatment such as being with their friends and being given stickers. (U)**^18^  “He just says he likes going on the van and having a sticker. [Interviewer: And he likes the sticker, do you know what he likes about going on the van?] I think it’s the chair, because they let him play with the chair.” ^(p. 89)^ |
| **A small number of children had reported to their parents that they did not like the taste or another aspect of treatment (e.g. that it was ‘gluey’) but, again, acceptability was often based on more than one factor. (U)**^18^  “That was it really just it didn’t taste very nice but she was delighted because she had stickers [laughs] you know I was like ‘What was it like, what did they do?’ and she said ‘They put something in my mouth and it didn’t taste very nice’”. ^(p. 89)^ |

| **Synthesised finding 4: Barriers that persist – 48 findings (48U)** |
| --- |
| **Category 1: Parental beliefs (n=28)** |
| **Participants’ opinions regarding the value of the program were a further indication of parental expectations. (U)**^26^  “The first visit though they don’t do much. They just look at the teeth and count them. I was thinking they did more like screening, and the first free dental visit is just to kind of help give you awareness and to show you, I think it’s more screening yeah and seeing where your kid’s at, seeing what potential things they would need.” ^(p. 4)^ |
| **Several parents seemed disappointed that so little was done during the appointment. (U)**^26^  “We snuck in one free visit, the under-three visit, and all they did was put her in a chair. There was no checking (…) So, there goes my free thing, like the freebie, and I haven’t been back since. And then I went last week and something’s growing and we have to go again tomorrow to see what we can do about it.” ^(p.4-5)^ |
| **One participant disagreed with the recommendation (age for the first visit), indicating that a child should have more than one tooth before visiting the dentist. (U)**^26^  “I think it depends on when they first start teething ’cause I got late teethers. One was ten and a half months, and the other one was eleven months, so he would have only had the one tooth.” When asked if that was not enough teeth to visit the dentist, she replied, “It depends, but I’d want to get him in maybe by two.” She then added, “But it’s not like it’ll hurt. It’s not like going to the dentist for your baby is going to be a waste of time. It’s always a good thing, so that you know that everything’s good.” ^(p. 3)^ |
| **Parents in the prevention arm found this strategy acceptable as long as the carious teeth were pain-free and considered it the less ‘radical’ or ‘significant’ method of treatment. (U)**^19^  “If she’d have been having a lot of pain, I’d have thought differently and I think … something more radical to either get rid of it, take the tooth out or to have a filling or whatever, so do something more significant rather than just the painting.” ^(p. 7)^ |
| **Parents raised concerns about the potential of further deterioration of their child’s teeth resulting in dental pain and/or affecting the child’s permanent successor tooth. (U)**^19^  “I’m all for that provided it doesn’t cause any more damage … My two concerns were A) … the decay was going to cause more damage and therefore she’s going to get some pain from it. And the second thing is whether it’s going to damage the adult teeth underneath …” ^(p.7)^ |
| **Some parents strongly believe that biological rather than behavioral factors contribute more to the development and progression of tooth decay. (U)**^16^  “Q: ‘Why do you think the decay has ceased?’ A: ‘It’s just the way her teeth are, it’s got nothing to do with the programme’”. ^(p.410)^ |
| **Mothers who considered themselves as experienced and trustworthy regarding motherhood did not impute importance to these actions. (U)**^20^  “I did not want to participate in the educational activities, because I think what they learned there, I already knew. Thus, I did not go to any”. ^(p. 672)^ |
| **Regardless of the number of children, the mothers interviewed perceived the educational actions as important only for preparing women who have not had experience with childcare. (U)**^20^  “I think there could be more meetings for mothers like my sister. She is young. She will be a mother for the first time. She has no experience. She does not know anything. So, I wanted more meetings for younger mothers”. ^(p. 672)^ |
| **Family members, friends, and especially their own mothers were important sources of information about childcare. (U)**^20^  “I learn from my mother. My mother is an encyclopedia. Because she raised five children and they are alive… You learn everything from your mother, and I recorded it”. ^(p .672)^ |
| **The mothers used a variety of hygiene materials for the oral health care of their children, the materials used were mainly determined by the family and social contexts. Some guidelines from health professionals were not incorporated into childcare because they conflicted with the mother's reality. (U)**^20^  “I have cleaned it with vinegar, a little bit of bicarbonate and water. Just a little bit of bicarbonate. Some days, I use a cloth to remove milk residue. My aunt taught me to do it like this”. ^(p. 672)^ |
| **Parents stated that general dentists might refuse to treat young children and the treatment by paediatric dentists is not affordable.** **(U)**^17^  “Dental check-ups are expensive. Just having a look is expensive. I observe my child’s teeth at home from time to time. If I cannot find any problems, I do not send him to see a dentist.” ^(p. 3)^ |
| **Majority of the parents reported that they used to get multiple opinions from different people to resolve the issues instead of consulting a dentist. (U)**^27^  “We do get many doubts regarding tooth problems. Most of the time, we tend to google it for the solutions. It is more comfortable and convenient”. ^(p. 219)^ |
| **Lack of awareness about the importance of primary teeth was one of the crucial determinants for child's dental visit. (U)**^27^  “Dental treatments are time-consuming, and it requires multiple visits. It is tough to leave our daily wages for the sake of a tooth which is not even permanent”. ^(p. 219)^ |
| **Time and commuting were the main problems against getting involved with the in-person components of the intervention, espe­cially face-to-face motivational interview (MI) sessions and MI follow-up calls. (U)**^21^  “It is very difficult for me to come here; I have to wake up early, and so does the child”. ^(p.7)^ |
| **Children who had no dental visit had plentiful oral problems. Most of the parents considered dental caries as the only oral health problem. On probing questions, other problems were mentioned.** **(U)**^27^  “Long back, she injured her front teeth while cycling. She made a fuss for some time and then she settled down. A couple of months later, the tooth turned yellow, and since she had no complaints, we left it untreated”. ^(p. 218)^ |
| **Most parents did not have adequate knowledge of preventive dental treatments. (U)**^27^  “Is it possible to prevent tooth decay? We never knew that there is an option of sealing the tooth! It seems to be better than having three, or four, or five fillings in the mouth. If we had known about this before, our son would have escaped the painful drills in his teeth”.  ^(p. 219)^ |
| **The conventional arm was described by some parents as the ‘traditional’ or ‘expected’ treatment based on their own experience; they were not aware of other options. (U)**^19^  “I think it was the filling, the traditional filling … ..I didn’t realise there were other treatment options to be fair. I had to get a filling, it was a needle… and I expected it before I was told about any other options”.^(p.4-5)^ |
| **Past dental experiences affected parents’ preferences and expectations of fillings and injections and it appeared that some parents re-lived their own childhood dental experiences when their child attended for dental treatment. (U)**^19^  “…. when I was little I was petrified because I had a bad experience. And obviously because my dentist weren’t as good as what they got now, I ended up losing my teeth on bottom...once you have a bad experience it puts you off it a little bit”. ^(p.5)^ |
| **Others brought their child because they had a toothache or noticed early signs of decay. (U)**^26^  “I have horrible teeth, so I wanted her to go and see. . . Just wanting her to be comfortable and getting used to going because I – not just bad teeth but a lot of anxiety around teeth. . . So, I wanted her to feel comfortable and not have my issues, both physically and mentally”. ^(p. 4)^ |
| **Some parents viewed the more invasive nature of the conventional approach for managing carious primary teeth negatively. (U)**^19^  “I wondered why they were gonna put her through that when this thing was going to fall out anyway …. why are you filling a tooth that’s gonna just fall out?”. ^(p.5)^ |
| **Being anxious of certain procedures because of potential pain, and concern that their child would not cooperate, was also reported by parents. (U)**^19^  “I think I were more nervous at first then she were … It was just the thought of her having an injection I thought, oh no it’s going to hurt … She’s not going to let them do it. But no, she were fine … ..he talked her through it. No problems at all”. ^(p.8-9)^ |
| **Parents reported being concerned about their child’s willingness to return for certain procedures. (U)**^19^  “And she’s playing with the drill [toy] but, like, if someone came near her mouth with the drill, she wouldn’t be happy at all. And I think we’d have had a lot more problems in getting her to sit down and keep coming back”. ^(p.9)^ |
| **Majority of the parents reported that their child's first dental visit was oriented to some complaints of teeth rather than a preventive check-up. They visited the dental clinic because of decayed teeth more specifically when it was symptomatic. (U)**^27^  “My child had decay in her teeth for a long period, but there were no complains. We made her clean the teeth twice, and it was all fine. Nevertheless, later there was swelling near the decayed teeth. We tried some home remedies, over the counter medicines, but it was not useful, and pain persisted. So, we visited the dentist”. ^(p. 217)^ |
| **The first experience for most of the children in the dental office was not very pleasant. In addition to the existing pain, the new environment added more stress to the children. This resulted in altered behaviour in children during their FDV. (U)**^27^  “The place was completely new to him. He was staring all around and questioning each and everything. The moment he was seated on the dental chair, he started crying and refused to open his mouth. They tried different methods to make him cooperative, but nothing helped. They had to restrain him completely to do the treatment”. ^(p. 217-8)^ |
| **All the parents agreed that they should have sought a more preventive approach before. (U)**^27^  “Doctor said that the teeth have to be removed as it was completely decayed. We were not much concerned about milk teeth and removed all four front teeth. However, when we noticed that he stopped smiling while taking photos because of his appearance, we felt very bad”. ^(p. 218)^ |
| **Some parents felt that when their child had prior knowledge of the specific procedures to be undertaken at the next dental visit that this would increase anxiety. (U)**^19^  “I think if they’re told they’re going for something, they get more worried … because I think if I’d just said we’ve got another check-up and then when she was in the chair, that would save her because she gets worked up way in advance”. ^(p.9)^ |
| **“Attitudes” to past treatment or the conventional dental treatment were also seen as important, with more than half of the parents reporting that their children had had bad past experiences with dental treatment, and many reporting that children had lost fillings. (U)**^25^  “… he was scared and didn’t like it (drilling)”. ^(p.15)^ |
| **A few parents had very little understanding or had completely forgotten the details of the study. (U)**^18^  “[Interviewer: Do you understand what the study is about?] Not really no”. ^(p. 98)^ |
| **Category 2: Difficulty in changing habits (n=9)** |
| **A number of parents felt that it was difficult to reduce their child’s consumption of sugary foods and drinks because the child would often misbehave if denied them. (U)**^16^  “He’ll say ‘mum can I have a chocolate?’, and I’ll say ‘yeah, you can have one’. But he wants a lot, and it depends on his day and his mood. Some days he’s just hard when he’s in a shitty mood. Sometimes he just doesn’t listen to me.” ^(p. 414)^ |
| **Some parents found it challenging to implement parental supervised brushing (PSB) at age over 1-year-old because they struggled to establish how far they could “push” their child while also keeping toothbrushing a positive experience. (U)**^15^  “I think it might be useful to give tips of how he might actually, he might move around or he might do this and try, like little tips of different sort of things that we could do in scenarios basically. Cause he wouldn’t really, he didn’t like it at the beginning and he would just bite and after a few months, it weren’t working he would just bite on the brush and now he kind of like, he’ll walk” around and play with the toothbrush and just put it in his mouth and bite on it and just do what he wants with it.” ^(p. 8)^ |
| **Some parents found implementing PSB to be challenging. Parents generally had very good intentions and saw the value of PSB. It was, on the other hand, sometimes deprioritised in the context of a busy family life with competing demands, such as getting older siblings to school on time. (U)**^15^  “…every evening we definitely do it. Most mornings I try do it after breakfast. Sometimes, if I’m in a massive rush to get to work and he’s going to nursery we don’t have time. But more often than not we do. Sometimes it’s a have breakfast, fly out the door situation you see…”. ^(p. 9)^ |
| **For some parents, the “Disney Magic Timer” app was viewed as burdensome and potentially negatively impacted on their child’s routine. (U)**^14^  “I didn’t find the App as useful for us. I can see why it could work for others. One, I’m quite strict on the devices, so we don’t generally allow their devices upstairs, and they definitely wouldn’t usually be allowed one in the bathroom […] Once she got the App, she wouldn’t do her teeth without the App, whereas she’d already been doing her teeth twice a day for her whole life [...] At one point we started to download it onto her iPad just purely so they could do her teeth, cause she wouldn’t do her teeth without it. But when I stopped using it, it was fine.” ^(p. 8)^ |
| **Downloading the “Disney Magic Timer” app conflicted with some parents views of allowing devices in the bathroom, and for others, the “Disney Magic Timer” app meant that it took longer for their child to brush their teeth, especially when a good routine had already been established. (U)**^14^  “But then as well every day when we got a sticker she wanted to go through all the stickers, so toothbrushing turned into a ten to fifteen minute job.” ^(p. 8)^ |
| **The parents' narrative suggests that children, similar to adults, struggle to make healthy food choices, despite knowing what these are. (U)^13^**  “He was brushing his teeth but … with all this fizzy drinks, he was my first child so, I just let him loose!” ^(p.5)^ |
| **Parents highlighted a need for other care environments to be aware of, and enforce appropriate dietary behaviours, such as schools (e.g., not provide sweet snacks after lunch), specifically as the child grows older and spends more time away from the parent. (U)^13^**  “...it does concern me at school because we restrict sugary snacks at home but school doesn’t and I have actually written to local council about this. They offer at lunch time like puddings and cakes as well as fruit as an alternative but we all know what the children are going to go for so it’s kind of a bit deflating that we restrict but he isn’t obviously restricted at school.” ^(p.6)^ |
| **Some parents, for example, felt their partners were not as supportive in maintaining optimal oral health for their children. (U)^13^**  “Saying that, going to the dentist hasn’t but now he’s getting older he’s getting lazy and it is a push to get him to do them. He will do them but, say if I’m at work and he’s at home with his Dad, guaranteed he won’t do his teeth.” ^(p.6)^ |
| **Some parents reported the difficulty in transitioning their children from flavoured infant toothpaste (of around 1000 ppm and usually sweet or mildly mint flavoured) to a child toothpaste (of up to 1500 ppm fluoride) due to the strong mint flavour. (U)^13^**  “I think they once had a go with ours but they found it too strong so, I bought a child’s […] I’ve never read it [fluoride content] to be honest.” ^(p.7)^ |
| **Category 3: Concerns regarding aesthetic results (n=11)** |
| **Some parents expressed concern with the black staining of lesions resulting from SDF treatment. The extent of concern varied depending on whether anterior or posterior teeth were treated.** **(U)**^23^  “I would [recommend] if it wasn’t the front teeth. Yeah, I would if they [stained teeth] weren’t visible.” ^(p. 4)^ |
| **Some parents expressed concern specifically with visible lesions on primary anterior teeth. (U)**^23^  “We don’t like that treatment because when my son laughs or smiles, his upper teeth look black and dirty, it’s not appropriate at all and the lower teeth are white.” ^(p.5)^ |
| **Some parents thought the carious lesions looked more severe because they turned black after SDF therapy. (U)**^17^  “I know the progression of tooth decay has been stopped by SDF treatment but it is black. It feels like the tooth decay is more severe than before because it used to be yellow or light brown.” ^(p.5)^ |
| **Parents believed that other people might admonish them about the black staining, thinking that they have taken no action about the carious lesions. (U)**^17^  “I do not like the black staining. People do not know it is staining and so they will consider it to be severe tooth decay. When people see the black staining, they say that the parents do not take good care of the child, like ‘why does her mother not help her to brush her teeth?’ or ‘why does her mother leave her decayed teeth there?’ They blame the parents for letting the tooth decay develop without taking any action. I have received a lot of pressure.” ^(p.5)^ |
| **Some parents accepted SDF therapy even though it caused black staining on the treated lesion because the appearance of dental caries already affects aesthetics, and the primary teeth will exfoliate. (U)**^17^  “The tooth decay already looked bad. It was black as well and the baby teeth will exfoliate anyway.” ^(p. 6)^ |
| **Parents whose children went through the SDF treatment shared that they had some feelings of fear and guilt about their children’s ECC. (U)**^24^  “A little bit, tiny bit I felt a little guilty because I was like, man they look like they just had a bunch of Oreo cookies. (…) But because I learned a little bit about [SDF treatment] it will be fine. It’s okay (…) I’ve seen other little kids with so much black stuff on their teeth and for my boys it’s just a few spots here and there. But I am glad that we did it for them.” ^(p. 6)^ |
| **Some parents felt that their children’s reaction might change to embarrassment once they are old enough to attend school. (U)**^23^  “[My child] is very young, he’s only 4 years old. He didn’t care about that yet. But it will be a big trouble when he goes to school, when he is at the age of going to school.” ^(p. 5)^ |
| **Parents raised an additional concern regarding the aesthetic aspect of PMCs in a child’s mouth as potentially being a visible sign of inadequacies in their parenting practices, but felt that if it was the best option then it was justified. (U)**^19^  “Obviously, I’m to blame as the Mum for the overall hygiene of his teeth. But you just felt like okay, everyone would see how bad mum I am … … But then I thought you know what, whatever is best for you. That’s what I was going to do”. ^(p.5)^ |
| **The ‘unnatural’ aesthetics of a PMC was a concern for some parents. There was concern that their child may become self-conscious about their appearance. (U)**^19^  “Slightly worried that with … all of her back teeth capped now … that like she’d notice, that other children didn’t … But she’s been absolutely fine. She’s not bothered by it”. ^(p.6)^ |
| **Some parents preferred a more restorative approach over prevention alone however they had reservations regarding the ‘unnatural’ appearance of PMCs. (U)**^19^  “I’d have been a bit iffy about probably leaving it and waiting and seeing, but I’m not quite sure how I feel about the stainless steel thing to be quite honest with you. I think I’d have preferred them to try and fill it rather than … you see it looks more natural”. ^(p.6)^ |
| **Some parents disfavoured PMCs for aesthetic reasons and thus favoured the preventive approach. (U)**^19^  “A silver cap on that tooth but we’ve at the moment decided not to … She wants one, I’m not sure. I think it’s me that’s saying no. I just (…) well, partly the aesthetics. I think having a piece of lump of silver in her mouth is not ideal at this age”. ^(p.7-8)^ |
